# Transposon-insertion sequencing screens unveil requirements for EHEC growth and intestinal colonization

**DOI:** 10.1371/journal.ppat.1007652

**Published:** 2019-08-12

**Authors:** Alyson R. Warr, Troy P. Hubbard, Diana Munera, Carlos J. Blondel, Pia Abel zur Wiesch, Sören Abel, Xiaoxue Wang, Brigid M. Davis, Matthew K. Waldor

**Affiliations:** 1 Division of Infectious Diseases, Brigham & Women’s Hospital, Boston, Massachusetts, United States of America; 2 Department of Microbiology, Harvard Medical School, Boston, Massachusetts, United States of America; 3 HHMI, Boston, Massachusetts, United States of America; University of Texas Southwestern Medical Center, UNITED STATES

## Abstract

Enterohemorrhagic *Escherichia coli* O157:H7 (EHEC) is an important food-borne pathogen that colonizes the colon. Transposon-insertion sequencing (TIS) was used to identify genes required for EHEC and *E*. *coli* K-12 growth *in vitro* and for EHEC growth *in vivo* in the infant rabbit colon. Surprisingly, many conserved loci contribute to EHEC’s but not to K-12’s growth *in vitro*. There was a restrictive bottleneck for EHEC colonization of the rabbit colon, which complicated identification of EHEC genes facilitating growth *in vivo*. Both a refined version of an existing analytic framework as well as PCA-based analysis were used to compensate for the effects of the infection bottleneck. These analyses confirmed that the EHEC LEE-encoded type III secretion apparatus is required for growth *in vivo* and revealed that only a few effectors are critical for *in vivo* fitness. Over 200 mutants not previously associated with EHEC survival/growth *in vivo* also appeared attenuated *in vivo*, and a subset of these putative *in vivo* fitness factors were validated. Some were found to contribute to efficient type-three secretion while others, including *tatABC*, *oxyR*, *envC*, *acrAB*, and *cvpA*, promote EHEC resistance to host-derived stresses. *cvpA* is also required for intestinal growth of several other enteric pathogens, and proved to be required for EHEC, *Vibrio cholerae* and *Vibrio parahaemolyticus* resistance to the bile salt deoxycholate, highlighting the important role of this previously uncharacterized protein in pathogen survival. Collectively, our findings provide a comprehensive framework for understanding EHEC growth in the intestine.

## Author summary

Enterohemorrhagic *E*. *coli* (EHEC) is an important food-borne pathogen that infects the colon. We created a dense EHEC transposon library and used transposon-insertion sequencing to identify the genes required for EHEC growth *in vitro* and *in vivo* in the infant rabbit colon. We found that there is a large infection bottleneck in the rabbit model of intestinal colonization and refined two analytic approaches to facilitate rigorous identification of new EHEC genes that promote fitness *in vivo*. Besides the known type III secretion system, more than 200 additional genes were found to contribute to EHEC survival and/or growth within the intestine. The requirement for some of these new *in vivo* fitness factors was confirmed, and their contributions to infection were investigated. This set of genes should be of considerable value for future studies elucidating the processes that enable the pathogen to proliferate *in vivo* and for design of new therapeutics.

## Introduction

Enterohemorrhagic Escherichia coli (EHEC) is an important food-borne pathogen that causes gastrointestinal (GI) infections worldwide. EHEC is a non-invasive pathogen that colonizes the human colon and gives rise to sporadic infections as well as large outbreaks [1–3]. The clinical consequences of EHEC infection range from mild diarrhea to hemorrhagic colitis and include the potentially lethal hemolytic uremic syndrome (HUS) [4,5].

The prototypical EHEC O157:H7 strain, EDL933, caused the first recognized EHEC outbreak in 1982 [6]. EDL933 has a 5.5 Mb chromosome and a 90 kb virulence plasmid [7,8]. The *E*. *coli* species pan-genome is large (>16,000 genes), and any given isolate contains a diverse, mosaic genome with approximately 1,500–2,000 conserved “core” genes [9–14] and an additional 3,000–4,000 “accessory” genes. The pathotype *E*. *coli* O157:H7 specifically contains one or more prophages encoding Shiga toxins and the Locus of Enterocyte Effacement (LEE) pathogenicity island [15]. These two horizontally acquired elements are critical EHEC virulence determinants. Shiga toxins contribute to diarrhea and the development of HUS [4,5,16]. The LEE encodes a type III secretion system (T3SS) and several secreted effectors. EHEC’s T3SS mediates attachment of the pathogen to colonic enterocytes, effacement of the brush border microvilli, and the formation of actin-rich pedestal-like structures underneath attached bacteria (reviewed in [17]). Once translocated into the host cell, T3SS effectors, which are encoded both inside and outside the LEE, target diverse signaling pathways and cellular processes [18,19]. A functional LEE T3SS is required for EHEC intestinal colonization in animal models as well as in humans [16,17,20–24].

In addition to the virulence factors that prompt the key symptoms of infection, EHEC also relies on bacterial factors that enable pathogen survival in and adaptation to the host environment. During colonization of the human GI tract, EHEC encounters multiple host barriers to infection, including but not limited to stomach acid, bile, and other host- and microbiota-derived compounds with antimicrobial properties (reviewed in [25]). EHEC is known to detect intestinal cues derived from the host and the microbiota to activate expression of virulence genes and to modulate gene expression both temporally and spatially [26–30]. However, a comprehensive, genome-wide analysis of bacterial factors that contribute to EHEC survival within the host has not been reported.

The development of transposon-insertion sequencing (TIS, also known as TnSeq, InSeq, TraDIS, or HITS) [31–34] facilitated high-throughput and genome-scale analyses of the genetic requirements for bacterial growth in different conditions, including in animal models of infection [35–44]. In this approach, the relative abundance of transposon-insertion mutants within transposon-insertion libraries provides insight into loci’s contributions to bacterial fitness in different environments [45,46]. Potential insertion sites for which corresponding insertion mutants are not recovered frequently correspond to regions of the genome that are required for bacterial growth (often termed “essential genes”), although the absence of a particular insertion mutant does not always reflect a critical role for the targeted locus in maintaining bacterial growth [47,48]. Comparative analyses of the abundance of mutants in an initial (input) library and after growth in a selective environment (e.g., an animal host) can be used to gauge loci’s contributions to fitness in the selective condition.

Here, transposon libraries were created in EHEC EDL933 and the laboratory-adapted *E*. *coli* K-12 and used to characterize their respective *in vitro* growth requirements. The EHEC library was also passaged through an infant rabbit model to identify genes required for intestinal colonization. Our data indicate that during infection of the GI tract, EHEC populations undergo a severe infection bottleneck that complicates identification of genes with true *in vivo* fitness defects. We used two complementary analytic approaches to mitigate the noise introduced by restrictive bottlenecks to identify over 200 genes required for efficient colonization of the rabbit colon. As expected, these included the LEE-encoded T3SS and *tir*, a LEE-encoded effector necessary for intestinal colonization [16,49]. In addition, 2 non-LEE effectors and many additional new genes that encode components of the bacterium’s metabolic pathways and stress response systems were found to enable bacterial colonization of the colon. Isogenic mutants for 17 loci, including *cvpA*, a gene necessary for intestinal colonization by diverse enteric pathogens [45,50,51], were constructed, validated in the infant rabbit model and tested *in vitro* under stress conditions that model host-derived challenges encountered within the GI tract. *cvpA* was found to be specifically required for resistance to the bile salt deoxycholate and therefore appears to be a previously unappreciated member of the bile-resistance repertoire of diverse enteric pathogens.

## Results/Discussion

### Identification of genes required for EHEC growth *in vitro*

**Fig 1:**
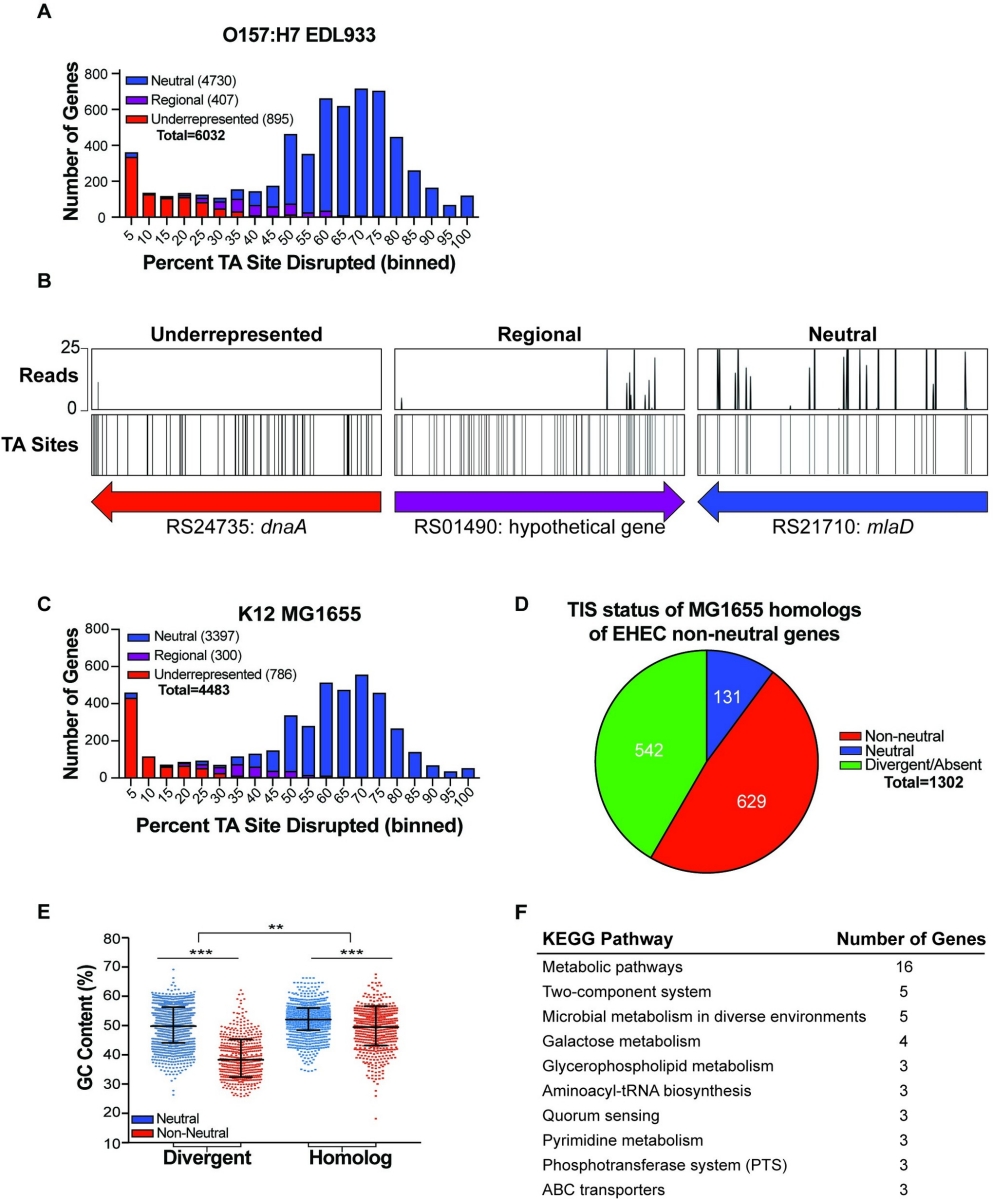
Analysis of essential genes in EHEC EDL933 and comparison to K-12 MG1655. (A) Distribution of percentage TA site disruption for all genes in EHEC EDL933. Genes are classified by EL-ARTIST as either underrepresented (red), regional (purple), or neutral (blue). (B) Transposon-insertion profiles of representative underrepresented, regional, and neutral genes. (C) Distribution of percentage TA site disruption for all genes in K-12 MG1655. Genes are classified by EL-ARTIST as underrepresented (red), regional (purple), or neutral (blue) using the same parameters as for the EDL933 library. (D) Genes classified as non-neutral in the ELD933 TIS library were compared to the MG1655 TIS library and categorized as either lacking a homolog (green), having the same classification in both libraries (red), or being non-neutral in EDL933 and neutral in MG1655 (blue). (E) GC content (%) of EDL933 genes with and without homologs in K-12 MG1655, classified by TIS classification (neutral or non-neutral). Neutral and non-neutral genes within each gene type (divergent or homolog) are compared using a Mann-Whitney U test with a Bonferroni correction. Ratios of neutral to non-neutral genes for each gene type are compared using a Fisher’s Exact Test; (**) indicates a p-value of <0.01 and (***) indicate a p-value of <0.001.(F) Top 10 KEGG pathways associated with genes that are non-neutral in EDL933 and neutral in MG1655.

The mariner-based Himar1 transposon, which inserts specifically at the TA dinucleotide [52] was used to generate a transposon-insertion library in EDL933. The library was characterized via high-throughput sequencing of genomic DNA flanking sites of transposon-insertion. To map the reads, we used the most recent EDL933 genome sequence [8] and annotation (NCBI, February 2017). Since this genome, unlike the initial EHEC genome [7], has not been linked to functional information (e.g., the EHEC KEGG database [53]), we generated a correspondence table in which the new gene annotations (RS locus tags) are linked to the original annotations (Z numbers) (S1 Table). This correspondence table enabled us to utilize historically valuable resources as well as the updated genomic sequence and should also benefit the EHEC research community. 137,805 distinct insertion mutants were identified, which corresponds to 52.5% of potential insertion mutants with an average of ~21 reads per genotype (S1 Fig). Sensitivity analysis revealed that nearly all mutants were represented within randomly selected read pools containing ~2 million reads. Increasing sequencing depth to ~3 million reads had a negligible effect on library complexity, suggesting that a sequencing depth of ~3 million reads is sufficient to identify virtually all genotypes within this EHEC library (S1 Fig). EHEC’s 6032 annotated genes were binned according to the percentage of disrupted TA sites within each gene, and the number of genes corresponding to each bin was plotted (Fig 1A).

As expected for a high-density Himar1 transposon-insertion library, which contains insertions at a majority of TA sites, this distribution was bimodal, with a minor peak comprised of genes disrupted in few potential insertion sites (Fig 1A, left), and a major peak comprised of genes that are disrupted in most or all potential insertion sites (Fig 1A, right) [46]. Based on the center of the major right-side peak, we estimate that ~70% of non-essential insertion sites have been disrupted in this EHEC library, a degree of complexity that enabled high-resolution analysis of transposon-insertion frequency.

Further analysis of insertion site distribution was performed using a hidden Markov model-based analysis pipeline (EL-ARTIST, see methods and [45]) that classifies loci with a low frequency of transposon-insertion across the entire coding sequence as ‘underrepresented’ (often referred to as ‘essential’ genes) or across a portion of the coding sequence as ‘regional’ (Fig 1B). All other loci are classified as ‘neutral’. Of EHEC’s 6032 genes, 895 genes were classified as underrepresented (red), 407 as regional (purple), and 4,730 as neutral (blue) (Fig 1A and 1B, S2 Table). Neutral genes are likely dispensable for growth in LB, whereas non-neutral genes (regional and underrepresented genes combined) likely have important functions for growth in this media or are otherwise refractory to transposon-insertion [47,48].

We identified Z Numbers (S1 Table) and the linked Clusters of Orthologous Groups (COG) [54,55] and KEGG pathways associated with the 1302 genes classified as non-neutral (underrepresented and regional) (S2 and S3 Tables). Each COG category was plotted against its “COG Enrichment Index”, which is calculated as the percentage of non-neutral genes in each COG category divided by the percent of the whole genome with that COG [56]. A subset of COGs, particularly translation, lipid and coenzyme metabolism, and cell wall biogenesis were associated with non-neutral genes at a frequency significantly higher than expected based on their genomic representation (S2 Fig). Collectively, the COG and a similar KEGG analysis (S3 Table) revealed that EHEC genes with non-neutral transposon-insertion profiles are associated with pathways and processes often linked to essential genes in other organisms [57].

### Evaluating the abundance of non-neutral genes in EDL933

Non-neutral genes comprise ~22% of EHEC’s annotated genes, a proportion of the genome that is substantially larger than the 8% and 9% observed in analogous TIS-based characterizations of *Vibrio parahaemolyticus* and *Vibrio cholerae* [45,50]. To evaluate whether the abundance of non-neutral loci was specific to EHEC or was characteristic of additional *E*. *coli* strains, a high-density transposon-insertion library was constructed in *E*. *coli* K-12 MG1655 [58]. EL-ARTIST analysis of the K-12 library (S1 Fig) was implemented with the same parameters as those for the EHEC library and classified 24% of genes as underrepresented (786 underrepresented, 300 regional and 3397 neutral; Fig 1C, S4 Table).

We compared gene classifications between homologous loci (see methods and S2 Table) and found a substantial concordance between the sets of genes with non-neutral insertion profiles: 83% (629/760) of the non-neutral EHEC genes with homologs in K-12 were likewise classified as non-neutral in K-12 (Fig 1D). Thus, analyses of non-neutral loci suggest either that most ancestral loci make similar contributions to the survival and/or proliferation of EHEC and K-12 in LB or that they are similarly resistant to transposon-insertion.

Previous analyses revealed that nucleoid binding proteins such as HNS, which binds to DNA with low GC content, can hinder Himar1 insertion [47]. Consistent with this observation, EHEC genes classified as non-neutral have a lower average GC content than genes classified as neutral (S2 Fig; blue vs red distributions). Interestingly, the disparity in GC content between neutral and non-neutral loci is particularly marked for EHEC genes that do not have a homolog in K-12 (divergent; Fig 1E). These analyses suggest that there is an association between GC content and transposon-insertion frequency in EHEC, as in other organisms, and that the prevalence of underrepresented loci among divergent loci may in part stem from the lower average GC content of these loci (S2 Fig). Additional studies are necessary to determine if the association between low GC content and reduced transposon-insertion is due to the binding of HNS or other nucleoid-associated proteins, or as yet unidentified fitness-independent transposon-insertion biases.

### Comparison of TIS and deletion-based gene classification

The sets of genes classified as underrepresented or regional in EHEC and K-12 transposon libraries were compared to the 300 genes classified as essential in the K-12 strain BW25113 based on their absence from a comprehensive library of single gene knockouts [59–61]. 98% of these genes (294/300) were also classified as underrepresented or regional in EDL933 (S2 Table) and MG1655 (S4 Table). The few loci previously classified as essential but not found to be underrepresented or regional in our analysis include several small genes, whose low number of TA sites hampers confident classification. One gene in this list, *kdsC*, was found to have insertions across the gene in both EDL933 and MG1655 (S2 Fig). *kdsC* knockouts have also been reported previously [62], confirming that this locus is not required for K-12 growth despite the absence of an associated mutant within the Keio collection. Thus, underrepresented and regional loci encompass, but are not limited to, loci previously classified as essential.

Several factors likely account for the frequent classification of “non-essential” loci as underrepresented or regional. First, loci can be classified as underrepresented even when viable mutants are clearly present within the insertion library (Fig 1A); insertions simply need to be consistently less abundant across a segment of the gene than insertions at other (neutral) sites. Loci may also be classified as underrepresented due to fitness-independent insertion biases, as discussed above [47,48]. Additional evidence that loci categorized as non-neutral by transposon-insertion studies are not necessarily essential for growth was provided by a recent study of essential genes in K-12 [63]. However, the more expansive non-neutral classification can provide insight into loci that enable optimal growth, in addition to those that are required.

### TIS-based comparison of EHEC and *E*. *coli in vitro* growth requirements

We further explored the 131 underrepresented EHEC loci (S5 Table) that were classified as neutral (able to sustain insertions) in K-12. Most of these genes are linked to KEGG pathways for metabolism, particularly metabolism of galactose, glycerophospholipid, and biosynthesis of secondary metabolites (S5 Table, Fig 1F). While this divergence could reflect the laboratory adaptation of the K-12 isolate, gene acquisition during EHEC evolution may have heightened the pathogen’s reliance on metabolic processes that are not critical for growth of K-12. Such ancestral genes may be useful targets for antimicrobial agents, as they might antagonize EHEC growth without disruption of closely related commensal Enterobacteriaceae populations. TIS studies in additional *E*. *coli* isolates are required to determine the relative contributions of the core and accessory *E*. *coli* genome to the list of essential genes.

### Identification of EHEC genes required for growth *in vivo*

To identify mutants deficient in their capacity to colonize the mammalian intestine, the EHEC transposon library was orogastrically inoculated into infant rabbits, an established model host for infection studies [16,49,64,65]. EHEC strain EDL933 causes diarrhea and similar pathology in infant rabbits as that previously described for EHEC strain 905 [16]. Transposon-insertion mutants were recovered from the colon 2 days post-infection, and the sites and abundance of transposon-insertion mutations were determined via sequencing. The relative abundance of individual transposon-insertion mutants in the library inoculum was compared to samples independently recovered from the colons of 7 animals to identify insertion mutants that were consistently less abundant in libraries recovered from the colon. Under ideal conditions, this signature is indicative of negative selection of the mutant during infection, reflecting that the disrupted locus is necessary for optimal growth within the intestine.

**Fig 2:**
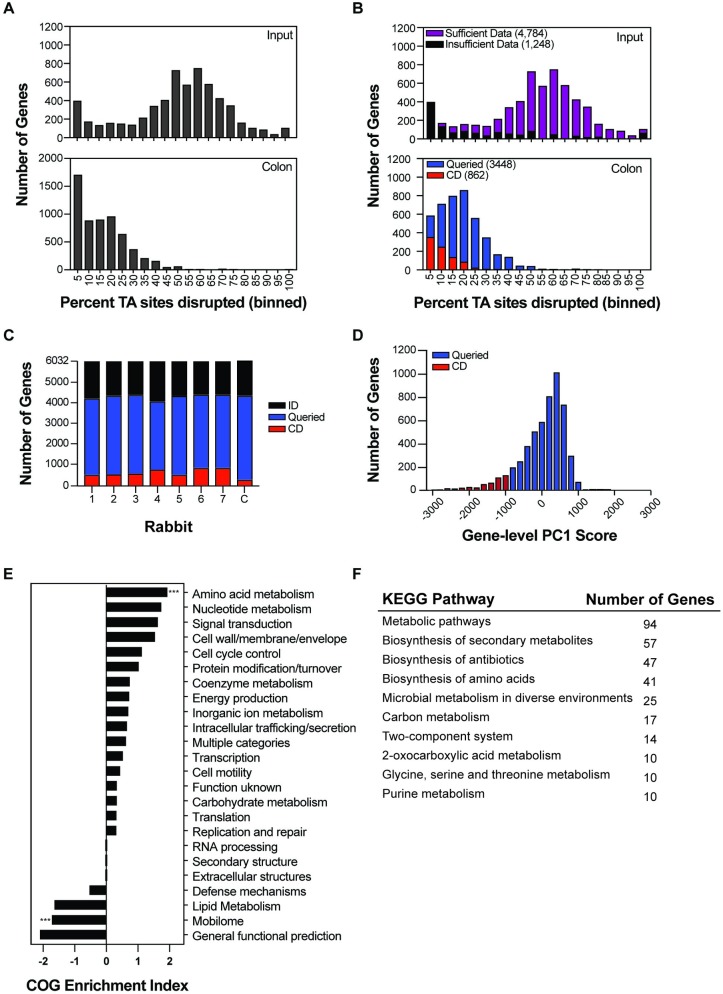
Identification of EHEC genes required for intestinal colonization. (A) Distribution of percentage TA site disruption for all genes in EDL933 in the library used to inoculate infant rabbits (top) and in a representative library recovered from a rabbit colon two days after infection (bottom). (B) Distribution of percentage TA site disruption for all genes in the inoculum library (top) and a representative library recovered from the rabbit colon (bottom) overlaid with the classifications from Con-ARTIST. Genes with insufficient data are removed from the bottom panel. (C) Distribution of Con-ARTIST gene classifications (insufficient data (ID, black), queried (blue), conditionally depleted (CD, red)) in each library recovered from seven infant rabbit colons two days post infection as compared to the inoculum (1–7). The last column, C, represents the consensus classification for all genes. To be classified as CD in this column, a gene needs to be classified as CD in 5 or more animals. (D) Distribution of PC1 scores across all EHEC genes. Red bins fall within the lowest 10% of PC1 scores (CD). Blue bins represent genes classified as queried (Q). (E) Conditionally depleted genes (as categorized by both Con-ARTIST and CompTIS; Group 1) by Clusters of Orthologous Groups (COG) classification. COG enrichment index (displayed as log_2_ enrichment) is calculated as the percentage of the CD genes assigned to a specific COG divided by the percentage of genes in that COG in the entire genome. A two-tailed Fisher’s exact test with a Bonferroni correction was used to test the null hypothesis that enrichment is independent of TIS classification. (***), p-value <0.0001. (F) Top 10 KEGG pathways of EHEC genes classified as conditionally depleted by both Con-ARTIST and CompTIS (Group 1).

Sequencing and sensitivity analyses of the 7 passaged libraries revealed that they contained substantially fewer unique insertion mutants than the library inoculum (23–38% total mutants recovered, ~30,000 of 120,000) (S1 Fig). These data are suggestive of population constrictions that could have arisen from 2 distinct but not mutually exclusive causes: 1) negative selection, leading to depletion of mutants deficient at *in vivo* survival or intestinal colonization; and/or 2) infection bottlenecks, population constrictions that lead to stochastic reductions in the average number of insertions per gene independent of genotype or selective pressures. We binned genes according to the percentage of TA sites disrupted within their sequences and plotted the number of genes corresponding to each bin for both the inoculum (Fig 2A-top) and a representative rabbit-passaged sample (Fig 2A-bottom). The passaged sample exhibited a marked leftward shift relative to the inoculum, a signature indicative of population constriction due to an infection bottleneck [46,66].

**Fig 3:**
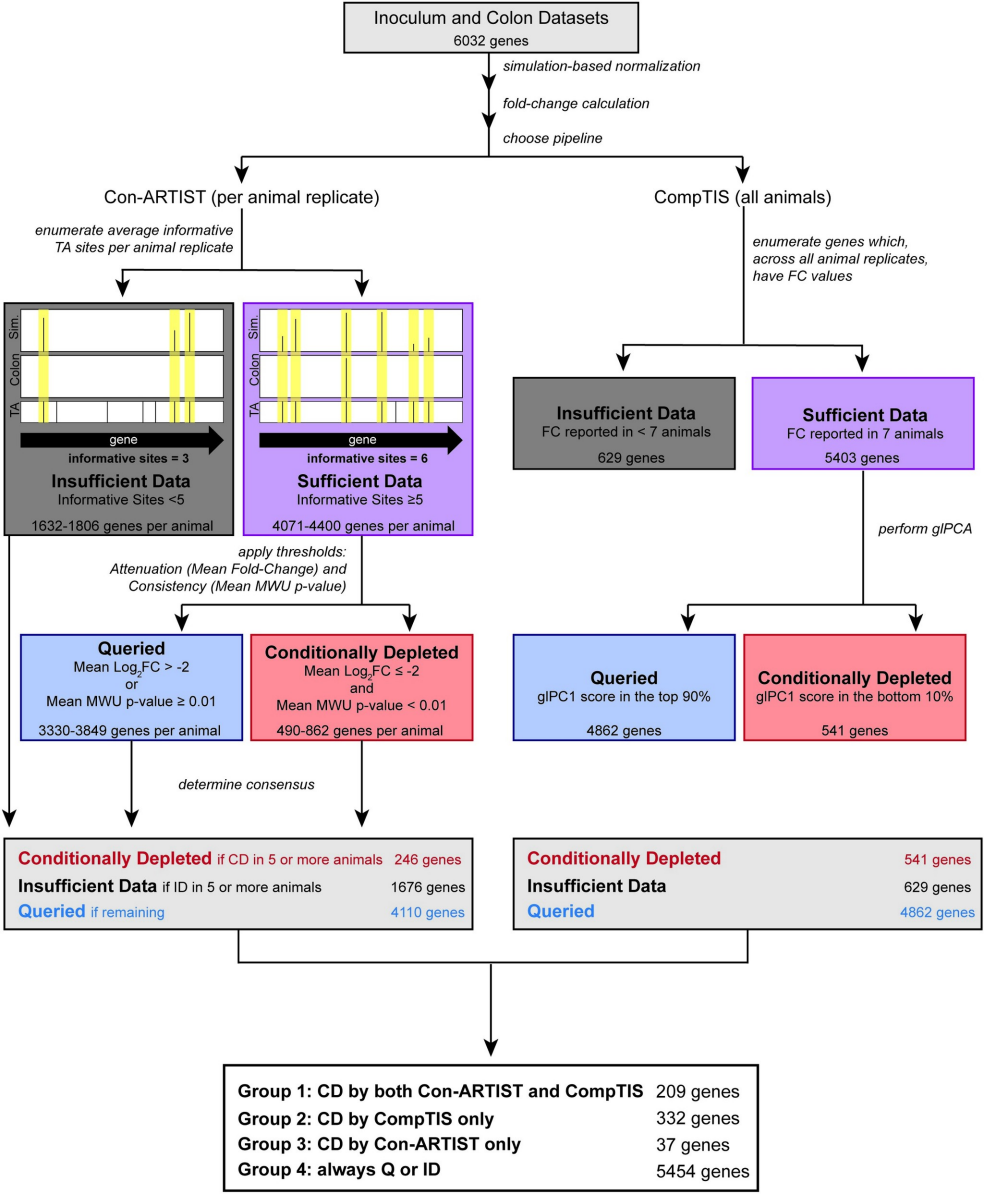
Schematic of analytic scheme to identify conditionally depleted genes. The TIS data was analyzed with Con-ARTIST and CompTIS. The first step in both pipelines is correction for bottleneck effects to facilitate identification of attenuated mutants. To do this, simulation-based normalization is performed on the inoculum dataset to model the stochastic loss observed in the colon datasets. Next, relative abundance of mutants (as represented by abundance of reads at each TA site) in the normalized inoculum and colon dataset were compared to determine a mean fold-change across each gene. Next, genes with mean fold-change values that are consistent with a signature of attenuation *in vivo* were identified. With Con-ARTIST, genes are first categorized based on their phenotype in each animal replicate, and then a consensus is determined for the phenotype across all replicates. To achieve this, in each animal, genes are first filtered by the number of informative sites—the number of unique TA sites between the input and output that have transposon-insertions. Genes with less than 5 informative sites are classified as insufficient data (ID, black). Genes with sufficient data (≥ 5 TA sites disrupted) are classified based on a dual standard of attenuation (mean log_2_ fold change ≤ -2) and consistency (Mann Whitney U p-value <0.01) as either queried (Q, blue) or conditionally depleted (CD, red). To choose genes with consistent phenotypes across the replicates, genes were classified as CD if they were classified as CD in 5 or more replicates. CompTIS synthesizes data across all animal replicates to identify genes important for colonization. Genes are first filtered to remove those without fold-change information across all replicates (ID, black). Gene-level PCA is performed on genes with sufficient data, and genes are classified as Q or CD based on their glPC1 score. Genes with a score in the bottom 10% of the glPC1 score distribution are considered to be attenuated (CD). The output of this workflow is 4 groups of genes summarized beneath the flowchart.

As infection bottlenecks can confound identification of genes with true fitness defects *in vivo* [45,66], we analyzed the TIS data using two complementary pipelines that mitigate the effects of bottlenecks: Con-ARTIST [45] and CompTIS [67]. Con-ARTIST was developed for this purpose [45], and we recently found that CompTIS, a PCA-based TIS analysis, is also useful for identifying genes whose inactivation leads to phenotypes that are consistent across animal replicates [67]. Both Con-ARTIST and CompTIS use iterative simulation-based normalization to compensate for experimental bottlenecks and facilitate discrimination between stochastic reductions in genotype abundance and reductions attributable to bona fide negative selection (mutants for which there was a fitness cost in the host environment); however, they use distinct methodologies to measure phenotypic consistency across animal replicates (see methods for details) in order to categorize genes as either conditionally depleted (CD), queried (Q), or insufficient data (ID) as compared to the inoculum library (Fig 3).

Con-ARTIST utilizes relatively stringent standards to identify robust candidate genes that facilitate EHEC intestinal growth in each rabbit. Such ‘conditionally depleted’ (CD) genes must contain sufficient transposon-insertions for Con-ARTIST analysis (at least 5 TA sites disrupted by transposon-insertion within the inoculum and output datasets) and meet a standard of a 4-fold reduction in read abundance (fold-change) that is consistent across TA sites in a gene, where consistency is measured using a Mann Whitney U (MWU) statistical test (Fig 3). Queried genes contain sufficient insertions for analysis but fail to meet the fold-change or p-value threshold. In contrast, genes classified as insufficient data (ID) have fewer than 5 TA sites disrupted by transposon-insertion. The output of gene categorization using these thresholds is displayed for a single rabbit in Fig 2B (additional animals in S3 Fig) and summarized for all animals in Fig 2C. An additional criterion that genes be classified as CD in 5 or more of the 7 animals analyzed was imposed to create a consensus list of CD genes (Fig 2C). In contrast to the >2000 genes classified as conditionally depleted in one or more animals, only 246 genes were classified as conditionally depleted across 5 or more animals (S6 Table).

CompTIS was also used to compare the seven libraries recovered from rabbit colons. CompTIS relies on PCA, a dimensional reduction approach used to describe the sources of variation in multivariate datasets, and can be used here as an alternative measure of phenotypic consistency between animals. This is particularly beneficial in this case, where the severe bottleneck limits the availability of individual transposon mutant replicates required for Con-ARTIST’s MWU p-value thresholds. In the CompTIS analysis, each gene’s fold change from the seven colon libraries was subjected to gene level PCA (glPCA) (see methods and [67]). Genes for which fold change information is not reported in all seven animal replicates are classified as ID. glPC1 describes most of the variation in the animals (S3 Fig) and represents a weighted average of the fold change values for each gene across the 7 animals (S6 Table). The signs and magnitudes of glPC1 were all similar (S3 Fig), indicating that each rabbit contributes approximately equally to glPC1, as expected for biological replicates. The distribution of glPC1 scores is continuous (Fig 2D) and describes each gene’s contribution to EHEC intestinal colonization. Most genes have a glPC1 score close to zero, suggesting that they do not contribute to colonization. However, the non-symmetrical distribution includes a marked left tail encompassing the lowest 10% of scores, which were classed as CD (Fig 2D, S3 Fig). The list of 541 CD genes includes nearly all (85%) of the genes classified as CD by the more conservative Con-ARTIST analysis outlined above (Fig 3).

These analyses yield four groups of genes (Fig 3). Group 1 includes the 209 genes categorized as CD by both ConARTIST and CompTIS and represents the highest confidence candidate genes required for colonization. Group 2 includes 332 genes identified as CD by CompTIS but classified as Q or ID by Con-ARTIST; it includes *ler* (glPC1 = -2290), a critical activator of the LEE T3SS [68], which is classified as queried by Con-ARTIST due to the relative paucity of unique insertion mutants. The lack of *ler* mutants is likely due to the small size of the gene (few TA sites) and to HNS binding occluding transposon insertion [68]. Group 3 includes the 37 genes identified as CD by ConARTIST but not by CompTIS, due to the absence of fold-change information for all seven replicate rabbits. Lastly, Group 4 includes the 5454 genes always identified as queried or insufficient data. Due to the severe bottleneck, we do not conclude that these genes are *not* attenuated relative to the wild type strain *in vivo*; it is likely that the list of CD loci called by either method is incomplete. Below, we primarily focused on Group 1 genes for further analysis, as they represent the most robust candidates for factors promoting EHEC intestinal colonization.

Using our Z correspondence table (S1 Table), 89% (186/209) of Group 1 genes were assigned to a COG functional category. CD genes were frequently associated with amino acid and nucleotide metabolism, signal transduction, and cell wall/envelope biogenesis, but only amino acid metabolism reached statistical significance after correction for multiple hypothesis testing (Fig 2E). These genes are also associated with KEGG metabolic pathways (particularly amino acid metabolism), several two-component systems, including *qseC*, which has previously been implicated in EHEC virulence gene regulation, and lipopolysaccharide biosynthesis [26] (S7 Table, Fig 2F). 30 of the 209 CD genes are EHEC specific, whereas the remaining 179 have homologs in K-12 (S6 Table), highlighting the importance of conserved metabolic pathways in the pathogen’s capacity to successfully colonize its colonic niche. Similar metabolic pathways were also found to be important for *V*. *cholerae* growth in the infant rabbit small intestine [45,69], raising the possibility of targeting metabolic pathways such as those for amino acid biosynthesis with antibiotics [70–72].

### Analyses of the requirement for T3SS and its associated effectors in colonization

**Fig 4:**
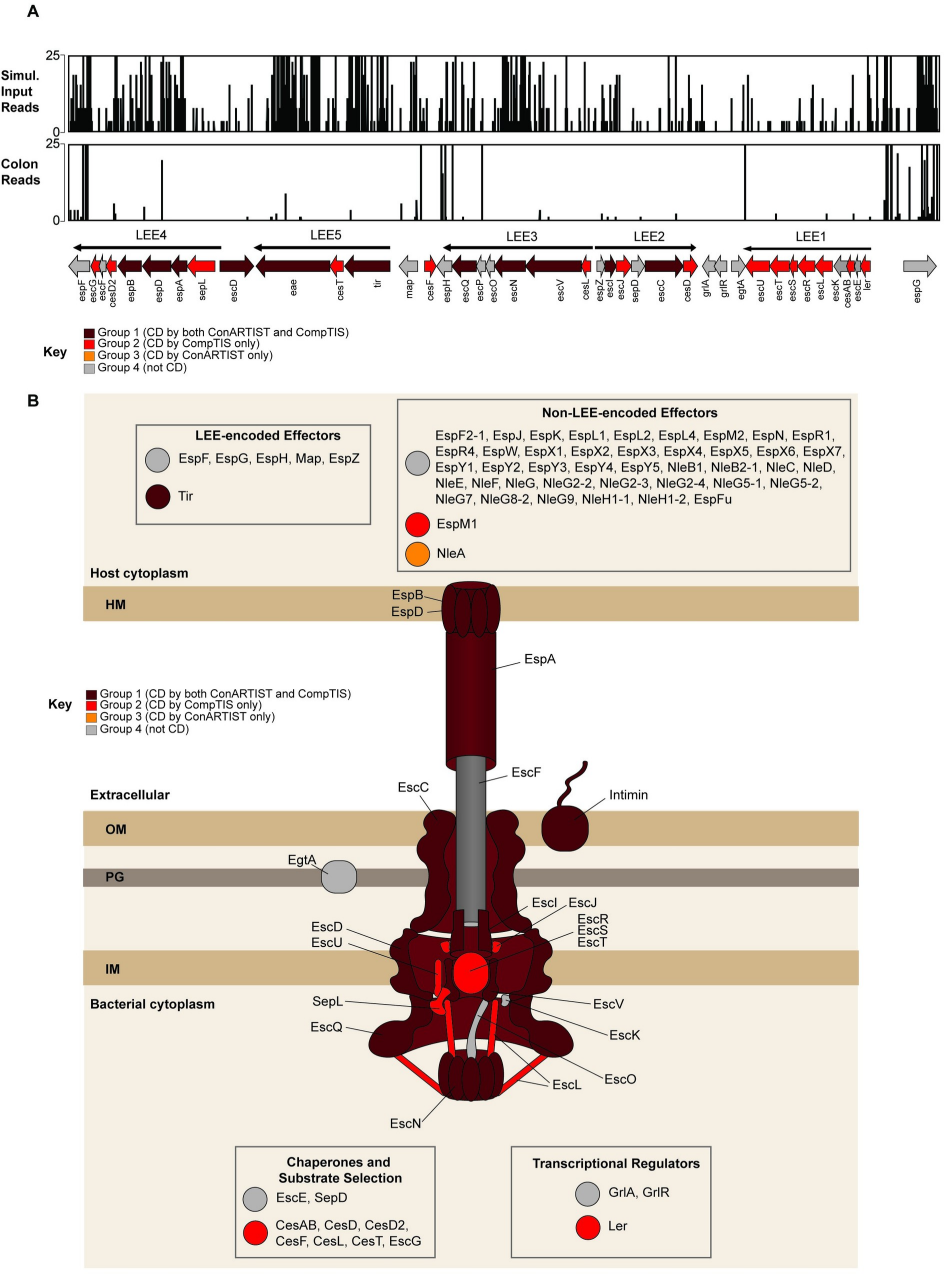
Con-ARTIST and CompTIS-based classification of LEE genes and T3SS effectors. (A) Artemis plots of reads in the LEE pathogenicity island in the control-simulated inoculum library (top) and a representative library recovered from the rabbit colon (middle). The genes in the LEE are displayed at the bottom. The key indicates which classification group each gene belongs: maroon genes belong to Group 1, which are classified as conditionally depleted (CD) by both Con-ARTIST and CompTIS; red genes belong to Group 2, which are classified as CD by CompTIS alone; orange genes belong to Group 3, which are classified as CD by Con-ARTIST alone; and gray genes belong to Group 4, which are not CD. (B) Schematic showing classification of the LEE genes and non-LEE-encoded effectors. Color key as above.

To assess the accuracy of our gene classifications using Con-ARTIST and CompTIS, we examined classifications within the LEE pathogenicity island, which encodes the EHEC T3SS and plays a critical role in intestinal colonization [16,20–23]. The LEE is comprised of 40 genes, including genes encoding the structural components of the T3SS, some of the pathogen’s effectors, their chaperones, and Intimin (*eae*), the adhesin that binds to the translocated Tir protein. In infant rabbits, previous studies using single deletion mutants revealed that *tir*, *eae*, and *escN*, the T3SS ATPase, were all required for colonization [16,49]. We observed a marked reduction in the abundance of insertions across nearly the entire LEE in the samples from the rabbit colons relative to the simulation-normalized input reads, indicating this locus is required for colonization (Fig 4A). However, the LEE has low GC content and is regulated through HNS binding [68] which when coupled with the infection bottleneck are expected to hamper assessment of gene contributions to intestinal fitness using Con-ARTIST alone.

12/40 LEE-encoded genes were categorized as Group 1 (CD by both ConARTIST and CompTIS), including 3 genes previously found to be required for colonization (*tir*, *eae* and *escN*) and 8 additional genes critical for T3SS activity, including translocon T3SS components (*espB*, *espD*, and *espA*) and structural components (*escD*, *escQ*, *escV*, *escI*, and *escC*) (Fig 4AB, S6 Table) [20,73–77]. Additionally, 15 genes were categorized as Group 2, including many encoding chaperones critical for T3SS assembly or T3SS structural components.

We also assessed the contribution of EHEC T3SS effectors on colonization. EHEC has 49 effectors: 6 genes encoded within the LEE (*espF*, *espG*, *espH*, *espZ*, and *map*) and 43 non-LEE encoded effectors (Nle). Nearly all effectors (5/6 LEE-encoded effectors and 41/43 Nle genes) were categorized into Group 4 (not CD). Consistent with these results, previous studies have shown that the LEE-encoded effectors *espG* and *map* are dispensable for robust colonization, and that Δ*espH* and Δ*espF* only had modest colonization defects [49]. The only effectors found to be important for colonization by either Con-ARTIST or CompTIS were *tir* (as expected) and 2 Nle genes: *nleA* and *espM1*. NleA was previously reported to be important for colonic colonization by a related enteric pathogen, *Citrobacter rodentium* [78], and is thought to suppress inflammasome activity [79]; EspM1 is thought to modulate host actin cytoskeletal dynamics [80,81]. Interestingly, mutants in *nleA* and *espM1* were also scored as attenuated in a TraDIS-based study of EDL933 growth in calves, where abundance of mutants in feces was analyzed [30]; however, this study used a much smaller miniTn5 library (covering 855 genes) and relied on a different analytic approach that used a less stringent definition of attenuation (solely based on 2-fold reduction in transposon mutant abundance in output vs input), making comparison to our study difficult. Additional studies are warranted to confirm and further explore how these 2 Nle effectors play pivotal roles promoting intestinal colonization.

### Validation of colonization defects in non-LEE encoded genes classified as conditionally depleted

We performed further studies of 17 conditionally depleted genes/operons that had not previously been demonstrated to promote EHEC intestinal colonization. All genes were part of Group 1, except *hupB*, which was identified as CD only by CompTIS (S6 Table). Mutants with in-frame deletions of either single loci (*agaR*, *cvpA*, *envC*, *htrA*, *hupB*, *mgtA*, *oxyR*, *prc*, *sspA*, *sufI*, *tolC*, and RS09610, a hypothetical gene of unknown function) or operons with one or more genes classified as conditionally depleted (*acrAB*, *clpPX*, *envZompR*, *phoPQ*, *tatABC*) were generated. Then, each mutant strain was barcoded with unique sequence tags integrated into a neutral locus in order to enable multiplexed analysis.

**Fig 5:**
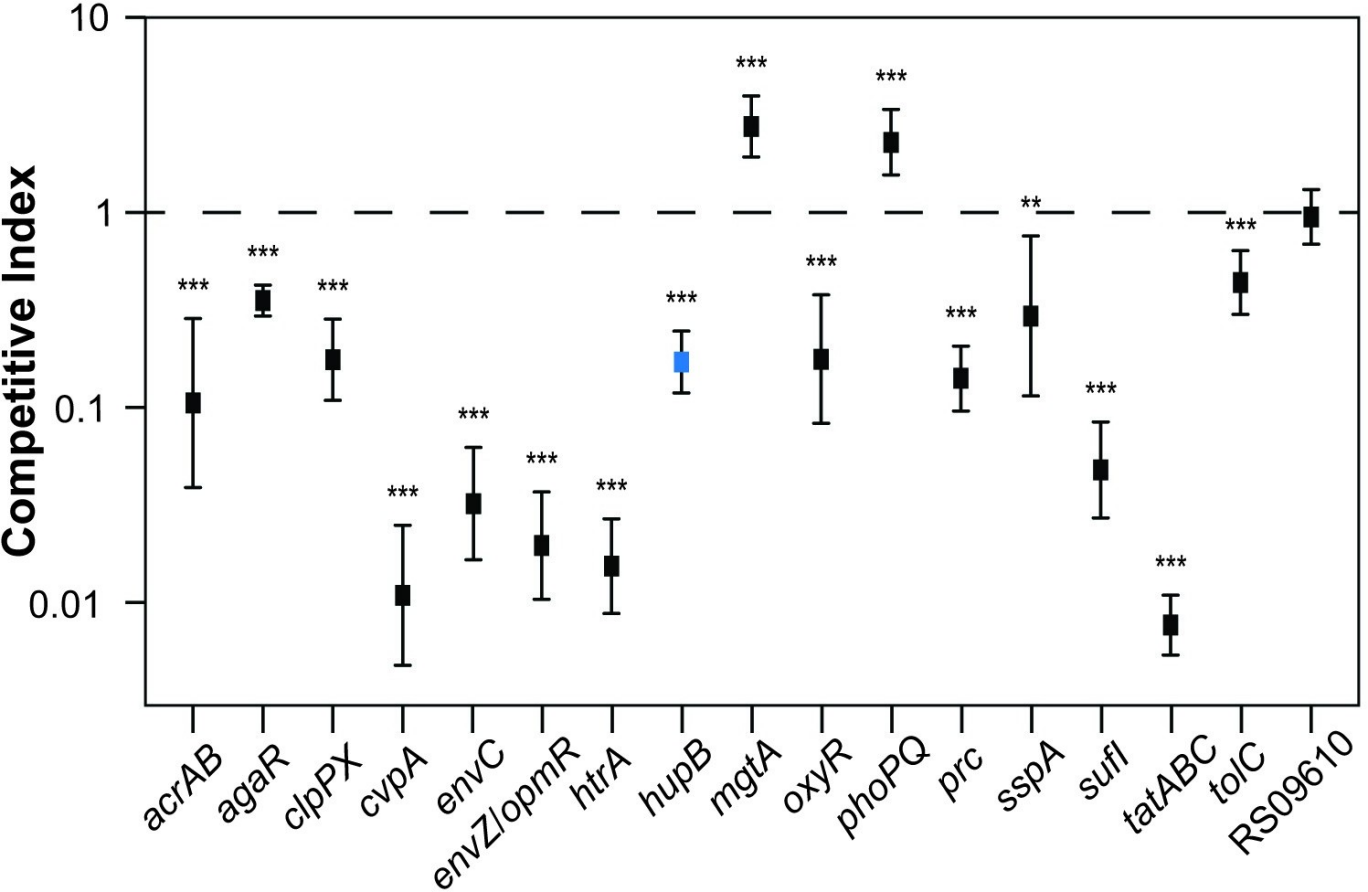
Validation of colonization defects in selected mutants. Competitive indices of indicated mutants vs wild type EHEC. Bar-coded mutants were co-inoculated with bar-coded wild-type EHEC into infant rabbits and recovered two days later from the colons of infected rabbits. Relative abundance of each mutant was determined by sequencing the barcodes. (**) p-value < 0.01 and (***) p-value <0.001. Δ*hupB*, which had a glPC1 score in the bottom 10%, but was not classified as CD by Con-ARTIST, is highlighted in blue.

The barcoded mutants, along with the barcoded WT EHEC, were co-inoculated into infant rabbits to compare the colonization properties of the mutants and WT. The relative frequencies of WT and mutant EHEC within colony-forming units (CFU) recovered from infected animals was enumerated by deep sequencing of barcodes, and these frequencies were used to calculate competitive indices (CI) for each mutant (i.e., relative abundance of mutant/WT tags in output normalized to input). 14 of the 17 mutants tested had CI values significantly lower than 1, validating the colonization defects inferred from the TIS data (Fig 5).

The *in vitro* growth of the barcoded mutants was indistinguishable from that of the WT strain (S4 Fig), suggesting that the *in vivo* attenuation is not explained by a generalized growth deficiency. In aggregate, these observations support our experimental and analytical approaches and provide confirmation that many of the genes classified as CD *in vivo* contribute to intestinal colonization.

### Many conditionally depleted loci exhibit reduced T3SS effector translocation and/or increased sensitivity to extracellular stressors

**Fig 6:**
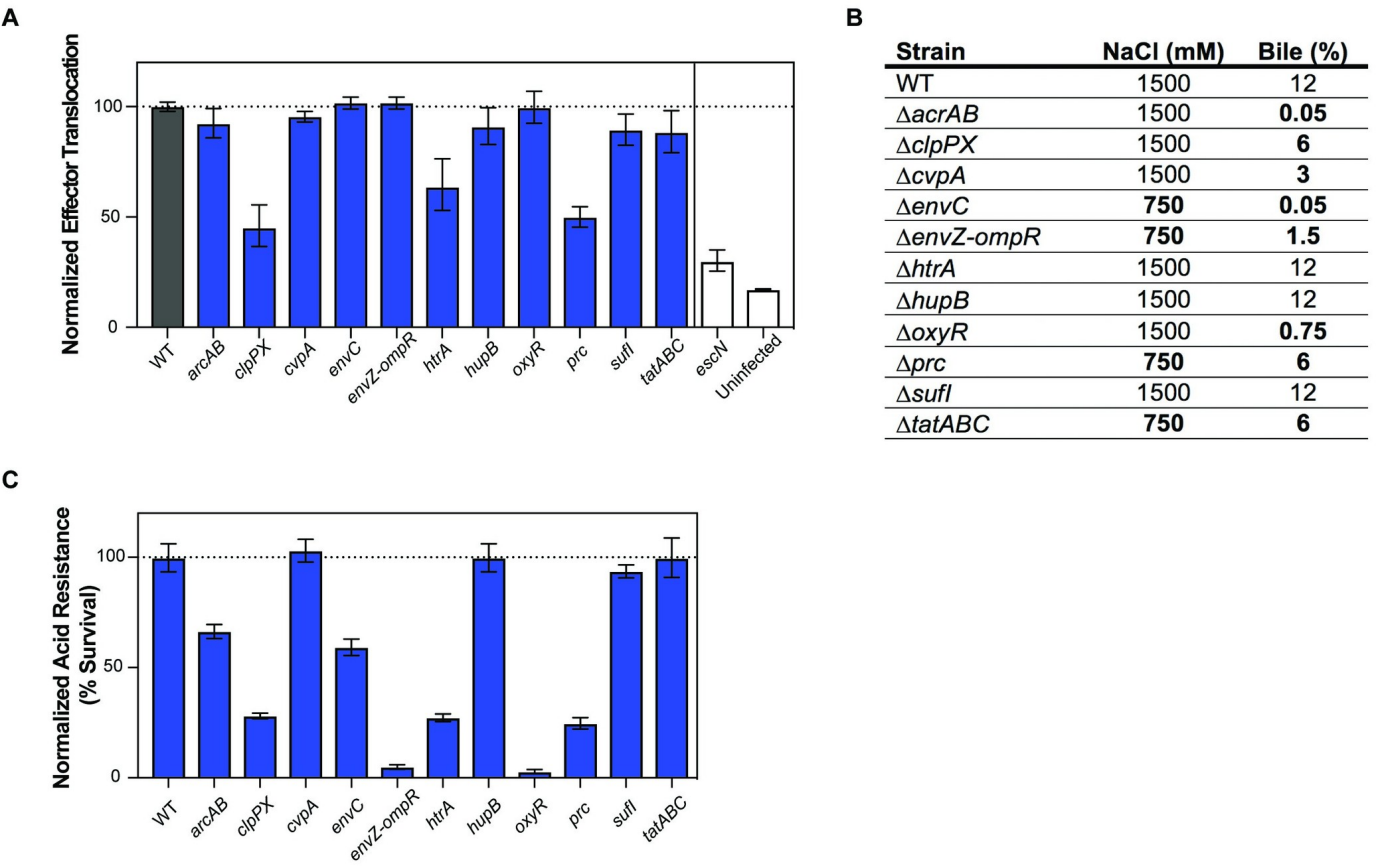
Effector translocation and survival in response to various gastro-intestinal stressors by mutants defective in colonization. (A) Normalized effector translocation of mutants compared to WT. Δ*escN*, a mutant that abrogates T3SS activity, was used as a control. Mutants were tested for their ability to translocate EspF-TEM1 into HeLa cells, as measured by a shift in emission spectra from 520 to 450 nm. Fluorescence was normalized to WT levels. Geometric means and geometric standard deviations are plotted. (B) MIC for NaCl (osmotic stress) and crude bile for the indicated mutants. Bold text highlights values differing from the wild-type. (C) Normalized acid resistance. Mutants were tested for their ability to survive low acid shock. Survival is shown as a percentage of the acid resistance of the WT. Geometric mean and geometric standard deviation are plotted.

The many new genes implicated in EHEC colonization by the TIS data could contribute to the pathogen’s survival and growth *in vivo* by a large variety of mechanisms. Given the pivotal role of EHEC’s T3SS in intestinal colonization, as well as previous observations that factors outside the LEE can regulate T3SS gene expression and/or activity (reviewed in [68]), we assessed whether T3SS function was impaired in the 11 mutants with CIs <0.3 (Fig 6A). Translocation of EspF (an effector protein) fused to a TEM-1 beta-lactamase reporter into HeLa cells was used as an indicator of T3SS functionality [82]. An Δ*escN* mutant, which lacks the ATPase required for T3SS function, was used as a negative control.

Deletions in three protease-encoded genes, *clpPX*, *htrA*, and *prc*, were associated with reduced EspF translocation (Fig 6A). Both ClpXP and HtrA have been implicated in T3SS expression/activity in previous reports [83–86]. The ClpXP protease controls LEE gene expression indirectly by degrading LEE-regulating proteins RpoS and GrlR [87]. The periplasmic protease HtrA (also known as DegP) has been implicated in post-translational regulation of T3SS as part of the Cpx-envelope stress response [85,86]. Interestingly, *prc*, which also encodes a periplasmic protease [87], also appears required for robust EspF translocation. Prc has been implicated in the maintenance of cell envelope integrity under low and high salt conditions in *E*. *coli* K-12 [88]. Consistent with this observation, in high osmolarity media a Δ*prc* EHEC mutant exhibited cell shape defects (S4 Fig). Deficiencies in the cell envelope associated with absence of Prc may impair T3SS assembly and/or function, perhaps also by triggering the Cpx-envelope stress response. Together, these observations suggest that *in vivo* these three proteases modulate T3SS expression/function, thereby promoting EHEC intestinal colonization.

We also investigated the capacity of each of the 11 mutant strains to survive challenge with three stressors–low pH, bile, and high salt (osmotic challenge)–that the pathogen may encounter in the gastrointestinal tract. Relative to the WT strain, all but one (*sufI*) of the mutant strains exhibited reduced survival following one or more of these challenges (Fig 6B and 6C), suggesting that exposure to these host environmental factors may contribute to the *in vivo* attenuation of these mutants. Many of the EHEC mutants exhibited sensitivities to external stressors that are consistent with previously described phenotypes in other organisms and experimental systems. For example, the EHEC Δ*acrAB* locus, which was associated with bile sensitivity in EHEC (Fig 6B), is known to contribute to a multidrug efflux system that can extrude bile salts, antibiotics, and detergents [89]. Our observation that mutants lacking the oxidative stress response gene *oxyR* are sensitive to bile and to acid pH is also concordant with previous reports linking both stimuli to oxidative stress [90–92]. Furthermore, the heightened sensitivity to bile, acid, and elevated osmolarity of EHEC lacking the two-component regulatory system EnvZ/OmpR is consistent with previous reports that EnvZ/OmpR is a critical determinant of membrane permeability, due to its regulation of outer membrane porins OmpF and OmpC. Mutations that activate this signaling system (in contrast to the deletions tested here) have been found to promote *E*. *coli* viability *in vivo* and to enhance resistance to bile salts [93].

The EHEC Δ*tatABC* mutant exhibited a marked colonization defect and a modest increase in bile sensitivity. The twin-arginine translocation (Tat) protein secretion system, which transports folded protein substrates across the cytoplasmic membrane (reviewed in [94,95]), has been implicated in the pathogenicity of a variety of Gram-negative pathogens, including enteric pathogens such as *Salmonella enterica* serovar Typhimurium [96–98], *Yersinia pseudotuberculosis* [99,100], *Campylobacter jejuni* [101], and *Vibrio cholerae* [102]. Attenuation of Tat mutants can reflect the combined absence of a variety of secreted factors. For example, the virulence defect of *S*. *enterica* Typhimurium *tat* mutants are likely due to cell envelope defects caused by the inability to secrete the periplasmic cell division proteins AmiA, AmiC and SufI [97]. Notably, single knock-outs of any of these genes does not cause *S*. *enterica* attenuation [97], but altogether their absence renders the cell-envelope defective and more sensitive to cell-envelope stressors, such as bile acids [98].

In EHEC, the Tat system has been implicated in Stx1 export [103], but because Stx1 was not a hit in our screen and is not thought to modulate intestinal colonization in infant rabbits [16], it is not likely to explain the marked colonization defect of the EHEC Δ*tatABC* mutant. The suite of EHEC Tat substrates has not been experimentally defined, although putative Tat substrates can be identified by a characteristic signal sequence [94,95]. A few substrates, including SufI, OsmY, OppA, MglB, and H7 flagellin, have been detected experimentally [103]. *sufI*, interestingly, was also a validated hit in our screen, and is the only CD gene that has a predicted Tat-secretion signal. However, the Δ*sufI* mutant did not display enhanced bile sensitivity, suggesting that attenuation of this mutant, and perhaps of the Δ*tatABC* mutant as well, reflects deficiencies in other processes. SufI is a periplasmic cell division protein that localizes to the divisome and may be important for maintaining divisome assembly during stress conditions [104,105]. *E*. *coli tat* mutants have septation defects [106], presumably from loss of SufI at the divisome. Interestingly, *envC*, another validated CD gene, encodes a septal murein hydrolase [107] that is required for cell division, and the Δ*envC* mutant also displayed increased bile sensitivity. Consistent with this hypothesis, in high osmolarity media, the Δ*sufI*, Δ*envC*, and Δ*tatABC* mutants exhibited septation or cell shape defects (S4 Fig). Collectively, these data suggest that an impaired capacity for cell division may reduce EHEC’s fitness for intraintestinal growth, and that at times this may reflect increased susceptibility to clearance by host factors such as bile.

### CvpA promotes EHEC resistance to deoxycholate

We further characterized EHEC Δ*cvpA* because other TIS-based studies of the requirements for colonization by diverse enteric pathogens (*Vibrio cholerae*, *Vibrio parahaemolyticus* and *Salmonella enterica* serovar Typhimurium) also classified *cvpA* as important for colonization, but did not explore the reasons for mutant attenuation [45,50,51]. *cvpA* has been linked to colicin V export in *E*. *coli* K-12 [108] as well as curli production and biofilm formation in UPEC [109]. The EHEC Δ*cvpA* mutant did not exhibit an obvious defect in biofilm formation or curli production (S5 Fig), suggesting that *cvpA* may have a distinct and previously unappreciated role in pathogenicity.

**Fig 7:**
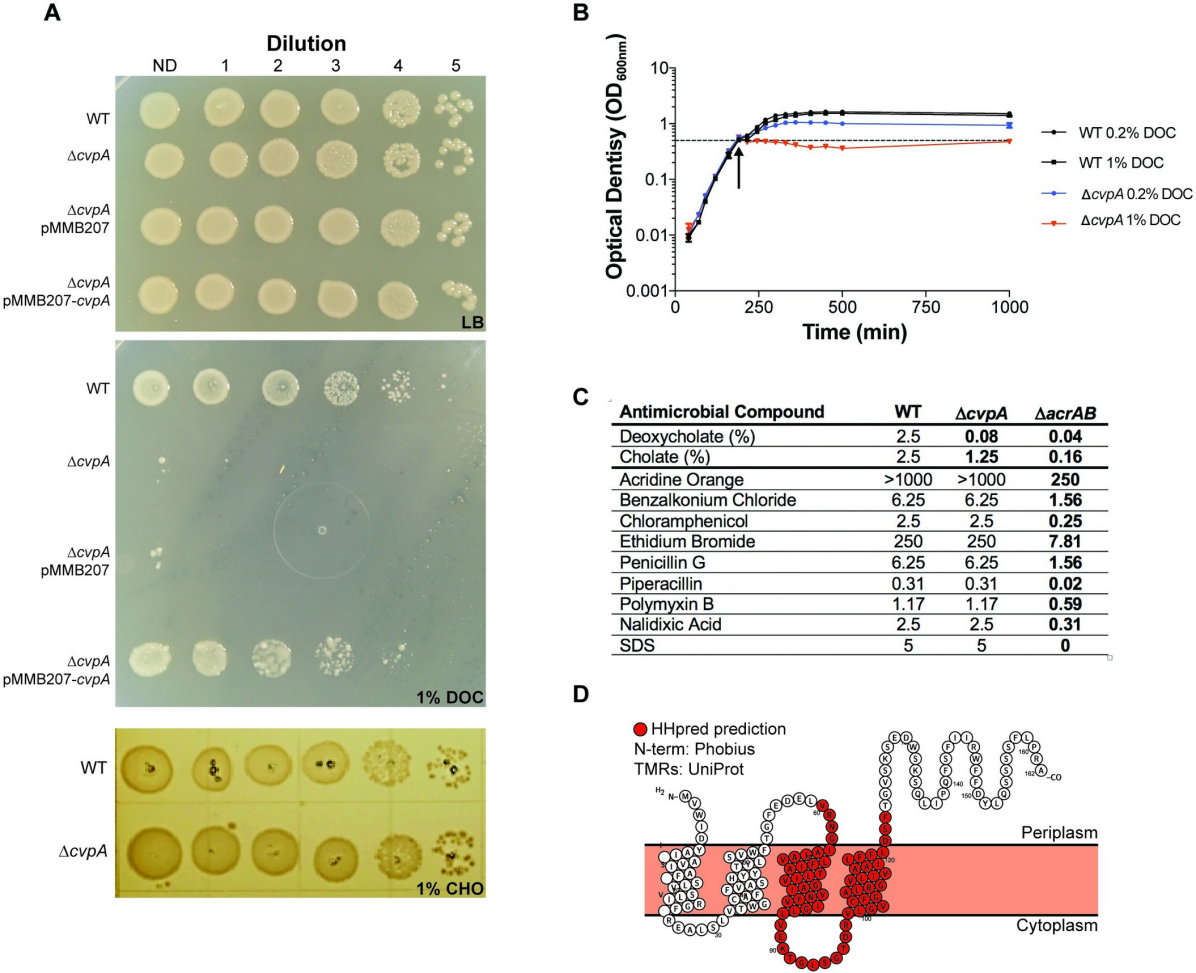
CvpA promotes EHEC resistance to deoxycholate. (A) Dilution series of WT, Δ*cvpA* mutant, and Δ*cvpA* mutant with arabinose-inducible *cvpA* complementation plasmid plated on LB, LB 1% cholate (CHO) or LB 1% deoxycholate (DOC). (B) Optical density of WT and Δ*cvpA* grown in LB and two concentrations of DOC, added at the indicated arrow. The average of three readings is plotted with errors bars indicating standard deviation. (C) MIC of antimicrobial compounds for WT and Δ*cvpA* and Δ*acrAB* mutants. Units are mg/mL unless specified otherwise. Bolded values are those different than the wild-type. (D) Schematic of predicted CvpA topology.

Initially, we confirmed that the Δ*cvpA* mutant exhibits an intestinal colonization defect in 1:1 competition vs the wild type strain (~20-fold defect, S5 Fig). To further characterize the sensitivity of the EHEC Δ*cvpA* mutant to bile (Fig 6B), we exposed the mutant to the two major bile salts found in the gastrointestinal tract, cholate (CHO) and deoxycholate (DOC) (Fig 7A and 7C) [90,110]. In contrast to WT EHEC, which displayed equivalent sensitivity to the two bile salts in MIC assays (MIC = 2.5% for both), the Δ*cvpA* mutant was much more sensitive to DOC than to CHO (MIC = 0.08% versus 1.25%). The Δ*cvpA* mutant’s sensitivity to deoxycholate was present both in liquid cultures and during growth on solid media (Fig 7A–7C).

*cvpA* lies upstream of the purine biosynthesis locus *purF*, and some Δ*cvpA* mutant phenotypes have been attributed to reduced expression of *purF* due to polar effects [108,111], but the growth of our Δ*cvpA* mutant was not impaired in the absence of exogenous purines (S5 Fig), suggesting the *cvpA* deletion does not adversely modify *purF* expression. Moreover, the DOC sensitivity phenotype of Δ*cvpA* was restored by plasmid-based expression of *cvpA*, confirming that this phenotype is linked to the absence of *cvpA* (Fig 7A).

Bile sensitivity has been associated with defects in the bacterial envelope or with reduced efflux capacity (reviewed in [110]). We assessed the growth of the Δ*cvpA* mutant in the presence of a variety of agents that perturb the cell envelope to assess the range of the defects associated with the absence of *cvpA*. The MICs of WT and Δ*cvpA* EHEC were compared to those of an Δ*acrAB* mutant, whose lack of a broad-spectrum efflux system provided a positive control for these assays. Notably, the Δ*cvpA* mutant did not exhibit enhanced sensitivity to any of the compounds tested other than bile salts. In marked contrast, the Δ*acrAB* mutant displayed increased sensitivity to all agents assayed (Fig 7C). These observations suggest that the sensitivity of the *cvpA* mutant to DOC is not likely attributable to a general cell envelope defect in this strain. *V*. *cholerae* and *V*. *parahaemolyticus* Δ*cvpA* mutants also exhibited sensitivity to DOC (S5 Fig), implying a similar role in bile resistance in these distantly related enteric pathogens.

A variety of bioinformatic algorithms (PSLPred, HHPred, Phobius, Phyre2) suggest that CvpA is an inner membrane protein with 4–5 transmembrane elements similar to small solute transporter proteins (Fig 7D). Phyre2 and HHPred reveal CvpA’s partial similarity to inner membrane transporters in the Major Facilitator Superfamily of transporters (MFS) and the small-conductance mechanosensitive channels family (MscC). The PFAM database groups CvpA (PF02674) in the LysE transporter superfamily (CL0292), a set of proteins known to enable solute export. In conjunction with findings presented above, these predictions raise the possibility that CvpA is important for the export of a limited set of substrates that includes DOC. Additional studies to confirm this hypothesis and to establish how CvpA enables export are warranted, particularly because this protein is widespread amongst enteric pathogens.

### Conclusions

Here, we created a highly saturated transposon library in EHEC EDL933 to identify the genes required for *in vitro* and *in vivo* growth of this important food-borne pathogen using TIS. This approach has transformed our capacity to rapidly and comprehensively assess the contribution an organism’s genes to growth in different environments [46,112,113]. However, technical and biologic issues can confound interpretation of genome-scale transposon-insertion profiles. For example, we found that EHEC genes with low GC content or those without homologs in K-12 were less likely to contain transposon-insertions (S2 Fig, Fig 1C), which may reflect processes other than reduced biological fitness. For example, the presence of nucleoid binding proteins could impair transposon-insertion into these loci. Unexpectedly, more than 100 of the genes conserved between EHEC and K-12 appear to promote the growth of the pathogen but not that of K-12 (S5 Table), suggesting that strain-specific processes may have enabled divergence of the metabolic roles of ancestral *E*. *coli* genes in these backgrounds. More comprehensive comparisons between additional *E*. *coli* isolates are needed to confirm this hypothesis.

In animal models of infection, bottlenecks that result in marked stochastic loss of transposon mutants can severely constrain TIS-based identification of genes required for *in vivo* growth. Analysis of the distributions of the EHEC transposon-insertions *in vitro* and *in vivo* (Fig 2) revealed that there is a large infection bottleneck in the infant rabbit model of EHEC colonization. Both Con-ARTIST, which applies conservative parameters to define conditionally depleted genes, and a PCA-based approach, CompTIS, were used to circumvent the analytical challenges posed by the severe EHEC infection bottleneck (Fig 3). These approaches should also be of use for similar bottlenecked data that often hampers interpretation of TIS-based infection studies. Validation studies, which showed that 14 of 17 genes classified as CD were attenuated for colonization (Fig 5), suggest that these approaches are useful. Besides the LEE-encoded T3SS, more than 200 additional genes were found to contribute to EHEC survival and/or growth within the intestine, most of which have never been linked to EHEC’s colonization capacity. This set of genes, particularly those involved in metabolic processes, should be of considerable value for future studies elucidating the processes that enable the pathogen to proliferate *in vivo* and for design of new therapeutics.

## Materials and methods

### Ethics statement

All animal experiments were conducted in accordance with the recommendations in the Guide for the Care and Use of Laboratory Animals of the National Institutes of Health and the Animal Welfare Act of the United States Department of Agriculture using protocols reviewed and approved by Brigham and Women’s Hospital Committee on Animals (Institutional Animal Care and Use Committee protocol number 2016N000334 and Animal Welfare Assurance of Compliance number A4752-01)

### Bacterial strains, plasmids and growth conditions

Strains, plasmids and primers used in this study are listed in S8 and S9 Tables. Strains were cultured in LB medium or on LB agar plates at 37°C unless otherwise specified. Antibiotics and supplements were used at the following concentrations: 20 μg/mL chloramphenicol (Cm), 50 μg/mL kanamycin (Km), 10 μg/mL gentamicin (Gm), 50 μg/mL carbenicillin (Cb), and 0.3 mM diaminopimelic acid (DAP).

A gentamicin-resistant mutant of *E*. *coli* O157:H7 EDL933 (Δ*lacI*::*aacC1*) and a chloramphenicol-resistant mutant of *E*. *coli* K-12 MG1655 (Δ*lacI*::*cat*) were used in this study for all experiments, and all mutations were constructed in these strain backgrounds except where specified otherwise. The Δ*lacI*::*aacC1* and Δ*lacI*::*cat* mutations were constructed by standard allelic exchange techniques [114] using a derivative of the suicide vector pCVD442 harboring a gentamicin resistance cassette amplified from strain TP997 (Addgene strain #13055) [115] or a chloramphenicol resistance cassette from plasmid pKD3 (Addgene plasmid #45604) [59] flanked by the 5’ and 3’ DNA regions of the *lacI* gene. Isogenic mutants of EDL933 Δ*lacI*::*aacC1* were also constructed by standard allelic exchange using derivatives of suicide vector pDM4 harboring DNA regions flanking the gene(s) targeted for deletion. *E*. *coli* MFDλpir [116] was used as the donor strain to deliver allelic exchange vectors into recipient strains by conjugation. Sequencing was used to confirm mutations.

A Δ*cvpA* strain was also constructed using standard allelic exchange in a streptomycin- resistant mutant (Sm^R^) of *V*. *parahaemolyticus* RIMD 2210633. A *cvpA*::tn mutant was used from a *Vibrio cholerae* C6706 arrayed transposon library [117].

### Eukaryotic cell line and growth conditions

HeLa cells were obtained from The Harvard Digestive Diseases Center (HDDC) Core Facilities at Boston Children’s Hospital and were cultured in Dulbecco’s modified Eagle’s medium (DMEM) supplemented with 10% fetal bovine serum (FBS). Cells were grown at 37°C with 5% CO_2_ and routinely passaged at 70 to 80% confluence; medium was replenished every 2 to 3 days.

### Transposon-insertion library construction

To create transposon-insertion mutant libraries in EHEC EDL933 Δ*lacI*::*aacC1*, conjugation was performed to transfer the transposon-containing suicide vector pSC189 [118] from a donor strain (*E*. *coli* MFDλpir) into the EDL933 recipient. Briefly, 100 μL of overnight cultures of donor and recipient were pelleted, washed with LB, and combined in 20 μL of LB. These conjugation mixtures were spotted onto a 0.45 μm HA filter (Millipore) on an LB agar plate and incubated at 37°C for 1 h. The filters were washed in 8 mL of LB and immediately spread across three 245x245 mm^2^ (Corning) LB-agar plates containing Gm and Kn. Plates were incubated at 37°C for 16 h and then individually scraped to collect colonies. Colonies were resuspended in LB and stored in 20% glycerol (v/v) at -80°C as three separate library stocks. The three libraries were pooled to perform essential genes analysis, and one library aliquot was used to as an inoculum for infant rabbit infection studies.

To create TIS mutant libraries in *E*. *coli* K-12 MG1655 Δ*lacI*::cat, conjugation was performed as above. 200 uL of overnight culture of the donor strain (*E*. *coli* MFDλpir carrying pSC189) and the recipient strain (MG1655 Δ*lacI*::cat) were pelleted, washed, combined and spotted on 0.45 μm HA filters at 37°C for 5.5 hours. Cells were collected from the filter, washed, plated on selective media (LB Km, Cm), and incubated overnight at 30°C. Colonies were resuspended in LB and frozen in 20% glycerol (v/v). An aliquot was thawed and gDNA isolated for analysis.

### Infant rabbit infection with EHEC transposon-insertion library

Mixed gender litters of 2-day-old New Zealand White infant rabbits were co-housed with a lactating mother (Charles River). To prepare the EHEC transposon-insertion library for infection of infant rabbits, 1 mL from one library aliquot was thawed and added to 20 mL of LB. After growing the culture for 3 h at 37°C with shaking, the OD_600_ was measured and 30 units of culture at OD_600_ = 1 (about 8 mL) were pelleted and resuspended in 10 mL PBS. Dilutions of the inoculum were plated on LB agar plates with Gm and Km for precise dose determination. An aliquot of the inoculum was saved for subsequent gDNA extraction and sequencing (input). Each infant rabbit was infected orogastrically with 500 μl of the inoculum (1x10^9^ CFU) using a size 4 French catheter. Following inoculation, the infant rabbits were monitored at least 2x/day for signs of illness and euthanized 2 days postinfection. The entire intestinal tract was removed from euthanatized rabbits, and sections of the mid-colon were removed and homogenized in 1 mL of sterile PBS using a minibeadbeater-16 (BioSpec Products, Inc.). 200 uL of tissue homogenate from the colon were plated on LB agar containing Gm and Km to recover viable transposon-insertion mutants. Plates were grown for 16 h at 37°C. The next day, colonies were scraped and resuspended in PBS. A 5 mL aliquot of cells was used for genomic DNA extraction and subsequent sequencing (Rabbits 1–7).

### Characterization of transposon-insertion libraries

Transposon-insertion libraries were characterized as described previously (50,119). Briefly, for each library, gDNA was isolated using the Wizard Genomic DNA extraction kit (Promega). gDNA was then fragmented to 400–600 bp by sonication (Covaris E220) and end repaired (Quick Blunting Kit, NEB). Transposon junctions were amplified from gDNA by PCR. PCR products were gel purified to isolate 200-500bp fragments. To estimate input and ensure equal multiplexing in downstream sequencing, purified PCR products were subjected to qPCR using primers against the Illumina P5 and P7 hybridization sequence. Equimolar DNA fragments for each library were combined and sequenced with a MiSeq. Reads for all TIS libraries have been deposited in the SRA database (Accession Number: PRJNA548905).

Reads were first trimmed of transposon and adaptor sequences using CLC Genomics Workbench (QIAGEN) and then mapped to *Escherichia coli* O157:H7 strain EDL933 (NCBI Accession Numbers: chromosome, NZ_CP008957.1; pO157 plasmid, NZ_CP008958.1) using Bowtie without allowing mismatches. Reads were discarded if they did not align to any TA sites, and reads that mapped to multiple TA sites were randomly distributed between the multiple sites. After mapping, sensitivity analysis was performed on each library to ensure adequate sequencing depth by sub-sampling reads and assessing how many unique transposon mutants were detected (S2 Fig). Next, the data was normalized for chromosomal replication biases and differences in sequencing depth using a LOESS correction of 100,000-bp and 10,000-bp windows for the chromosome and plasmid, respectively. The number of reads at each TA site was tallied and binned by gene and the percentage of disrupted TA sites was calculated. Genes were binned by percentage of TA sites disrupted (Fig 1A and 1C).

For essential gene analysis, EL-ARTIST was used as in [50]. Protein-coding genes, RNA-coding genes, and pseudogenes were included in this analysis. Briefly, EL-ARTIST classifies genes into one of three categories (underrepresented, regional, or neutral), based on their transposon-insertion profile. Classifications are obtained using a hidden Markov model (HMM) analysis following sliding window (SW) training (p <0.05, 10 TA sites). Insertion-profiles for example genes were visualized with Artemis.

For identification of mutants conditionally depleted in the rabbit colon as compared to the input inoculum, Con-ARTIST was used as in [119]. First, the input library was normalized to simulate the severity of the bottleneck as observed in the libraries recovered from rabbit colons using multinomial distribution-based random sampling (n = 100). Next, a modified version of the Mann-Whitney U (MWU) function was applied to compare these 100 simulated control data sets to the libraries recovered from the rabbit colon. All genes were analyzed, but classification as “conditionally depleted” (CD) was restricted to genes that had sufficient data (≥5 informative TA sites), met our standard of attenuation (mean log_2_ fold change ≤ -2), met our standard of phenotypic consistency (MWU p-value of ≤0.01), and had a consensus classification in 5 or more of the 7 animals analyzed. Genes with ≥5 informative TA sites that fail to exceed both standards of attenuation and consistency are classified as “queried” (Q, blue), whereas genes with less than 5 informative TA sites are classified as “insufficient data” (ID).

Gene-level PCA (glPCA) was performed using CompTIS, a principal component analysis-based TIS pipeline, as described in [67]. Briefly, log_2_ fold change values were derived by comparing read abundance in each sample to 100 control-simulated datasets as in Con-ARTIST. These fold change values were weighted to minimize noise due to variability (for details, see [67]). Next, genes that did not have a fold change reported for all 7 animals were discarded. The fold change values were then z-score normalized. Weighted PCA was performed in Matlab (Mathworks) with the PCA algorithm (pca).

Genes were categorized into 4 groups based on their classifications in Con-ARTIST and CompTIS (Fig 3).

### GC content

The GC content of classified genes was compared using a Mann-Whitney U statistical test and a Bonferroni correction for multiple hypothesis correction when more than one comparison was made. A p-value <0.05 was considered significant for one comparison, p<0.025 for two. A Fisher’s exact two-tailed t-test was used to compare ratios of classifications between groups, where a p-value of <0.01 was considered significant.

### In vivo competitive infection: pooled mutants versus wild-type

Barcodes were introduced into Δ*lacI*::*aacC1*and isogenic mutant strains as described previously [50,120]. Briefly, a 991bp fragment of *cynX* (RS02015) that included 51bp of the intergenic region between *cynX* and *lacA* (RS02020) was amplified using primers that contained a 30 bp stretch of random sequence and cloned into SacI and XbaI digested pGP704. The resulting pSoA176.mix was transformed into *E*. *coli* MFDλpir. Individual colonies carrying unique tag sequences were isolated and used as donors to deliver pSoA176 barcoded derivatives to EDL933 Δ*lacI*::*aacC1*and each isogenic mutant strain. Three barcodes were independently integrated into EDL933 Δ*lacI*::*aacC1*, and three barcodes into each isogenic mutant via homologous recombination in the intergenic region between *cynX* and *lacA*, which tolerates transposon-insertion *in vitro* and *in vivo*, indicating this locus is neutral for the fitness of the bacteria. Correct insertion of barcodes was confirmed by PCR and sequencing.

To prepare the culture of mixed EHEC-barcoded strains for the multi-coinfection experiment, 100 μl of overnight cultures of the barcoded strains were mixed in a flask and 1 mL of this mix was added to 20 mL LB. After growing the culture for 3 h at 37°C with shaking, the OD_600_ was measured and 30 units of culture at OD_600_ = 1 (about 8 mL) were pelleted and resuspended in 10 mL PBS. Dilutions of the inoculum were plated on LB agar plates with Gm and Cb for precise CFU determination. 10 infant rabbits were inoculated and monitored as described above, and colon samples collected. Tissue homogenate was plated, and CFU were collected the following day. gDNA was extracted and prepared for sequencing as in [120].

The quantification of sequence tags was done as described previously [120]. In brief, sequence tags were amplified from the inoculum culture and libraries recovered from rabbit colons. The relative *in vivo* fitness of each mutant was assessed by calculating the competitive index (CI) as follows:

We compare two strains (Δ*lacI*::*aacC1* and isogenic mutant) in a population with frequencies *f*_*wt*_ and *f*_*mut*,*x*_ respectively where x is one of 17 mutant strains with a deletion in gene x. For simplicity, we assume here that both expand exponentially from a time point t_0_ to a sampling time point t_s_, their relative fitness (offspring/generation) is proportional to the competitive index CI: ln(fmut,x,sfmut,x,0fwt,sfwt,0)=ln(CI). Here, *f*_*wt*,*0*_ and *f*_*mut*,*x*,*0*_ are the frequencies of the strains in the inoculum, measured in triplicates, and *f*_*wt*,*s*_ and *f*_*mut*,*x*,*s*_ describe the frequencies at the sampling time point in the animal host. Because the WT strain was tagged with 3 individual tags and the inoculum was measured in triplicate, we have 3x3 = 9 measurements of the ratio fwt,sfwt,0. The same is true for all mutant strains, such that we have 9 measurements of the ratio fmut,x,sfmut,x,0. In total, we therefore have 3x3x3x3 = 81 CI measurements for each mutant per animal. To determine intra-host variance in these 81 measurements, a 95% confidence interval of the CI in single animal hosts was determined by bootstrapping. For combining the CIs measured across all 10 animal hosts, we performed a random-effects meta-analysis using the metafor package [121] in the statistical software package R (version 3.0.2). The pooled rate proportions and 95% confidence intervals were calculated using the estimates and the variance of CIs in each animal determined by bootstrapping and corrected for multiple testing using the Benjamini-Hochberg procedure.

### In vitro growth

Each bacterial strain was grown at 37°C overnight. The next day, cultures were diluted 1:1000 into 100 uL of LB in 96-well growth curve plates in triplicate. Plates were left shaking at 37°C for 10–24 hours. Absorbance readings at 600nm were normalized to a blank, and the average of each triplicate was taken as the optical density.

### T3SS translocation assays

T3SS functionality was assessed by translocation of the known EHEC T3SS effector protein EspF into HeLa cells as described previously [77]. Briefly, the plasmid encoding the effector protein EspF fused to TEM-1 beta-lactamase was transformed into each of the bacterial strains to be tested. Overnight cultures of each bacterial strain were diluted 1/50 in DMEM supplemented with HEPES (25mM), 10% FBS and L-glutamine (2mM) and incubated statically at 37°C with 5% CO2 for two hours. This media is known to induce T3SS expression [122]. HeLa cells were seeded at a density of 2x10^4^ cells in 96-well clear bottom black plates and infected for 30 minutes at an MOI of 100. After 30 minutes of infection IPTG was added at a final concentration of 1mM to induce the plasmid-encoded T3SS effector. After an additional hour of incubation, monolayers were washed in HBSS solution and loaded with fluorescent substrate CCF2/AM solution (Invitrogen) as recommended by the manufacturer. After 90 minutes, fluorescence was quantified in a plate fluorescence reader with excitation at 410nm and emission was detected at 450nm. Translocation was expressed as the emission ratio at 450/520nm to normalize beta-lactamase activity to cell loading and the number of cells presented at each well, and then normalized to WT levels of translocation.

### Biofilm, curli production, and purine assays

Biofilm and curli production assays were performed as described previously [109]. For biofilm assays, bacterial cultures were grown in yeast extract-Casamino Acids (YESCA) medium until they reached an OD_600_ ~ 0.5 and 1/1000 dilution of this culture was used to seed 96-well PVC plates. The cultures were grown at 30°C for 48 hours and biofilm production was quantitatively measured using crystal violet staining and absorbance reading at 595nm. Relative biofilm production was normalized to the average of three WT samples. A two-tailed Mann Whitney U test was used to determine if biofilm production was significantly different. A p-value < 0.05 was considered to be significant. To test curli production, bacterial cultures were grown in YESCA medium until they reached an OD_600_ ~ 0.5 and then were struck to single colonies onto YESCA agar plates supplemented with Congo Red. Red colonies indicate curli production. To test if our Δ*cvpA* deletion had polar effects on *purF*, the mutant and WT were struck onto minimal media lacking exogenous purines.

### Acid shock assays

An adaptation of the acid shock method described in [123] was performed. Briefly, bacterial cultures were grown until mid-exponential phase (OD_600_ ~ 0.6), then diluted 20-fold in LB pH 5.5 and incubated for 1 hour before preparing serial dilutions and plating each culture to determine the relative percentage of survival in comparison to the wild-type EDL933 strain. The pH of the LB broth was adjusted using sterilized 1mM HCl and buffered with 10% MES. Values are expressed as percent survival normalized to WT.

### MIC assays

MIC assays were performed using an adaptation of a standard methodology with exponential-phase cultures [124]. Briefly, the different compounds to be tested (see Figs 6B and 7C) were prepared in serial 2-fold dilutions in 50 ul of LB in broth in a 96-well plate format. To each well was added 50 ul of a culture prepared by diluting an overnight culture 1,000-fold into fresh LB broth, growing it for 1 h at 37°C, and again diluting it 1,000-fold into fresh medium. The plates were then incubated without shaking for 24 h at 37°C.

### Bile salts survival assays

Bile salt sensitivity assays were adapted from [125]. For plate sensitivity assays, each bacterial strain was grown at 37°C until they reached mid-exponential phase of growth (OD_600nm_ of 0.5) and the culture was serially diluted and spot-titered onto LB agar plates supplemented with either 1% DOC or 1% CHO. Spots were air dried and plates incubated at 37°C for 24 h. For sensitivity assays done in liquid culture, each bacterial strain was grown at 37°C until it reached mid-exponential phase of growth (OD_600nm_ of 0.5) and then cultures were split and supplemented with either DOC, CHO or buffer (PBS) and bacterial growth was assessed by absorbance at 600nm.

### Growth in high-salt media

Bacterial strains were grown in either LB or LB supplemented with 0.3M NaCl until mid-exponential phase and analyzed by phase microscopy at 100x magnification.

### 1:1 competitive infection of Δ*cvpA* and wild-type

To test the Δ*cvpA in vivo* fitness defect, we competed Δ*cvpA* against a Δ*lacZ* mutant in the infant rabbits. To prepare the cultures for infection, 100 μl of overnight cultures of each strain were inoculated into 100 mL of LB Gm for. After 3 hours of growth at 37°C with shaking, the OD_600_ was measured and 30 units of culture at OD_600_ = 1 were pelleted and resuspend in 10 mL PBS. Then, 5 mL of the Δ*lacZ* culture was combined with 5 mL Δ*cvpA* to make a 1:1 mixture. Dilutions of the inoculum were plated on LB agar plates containing Gm and X-Gal (60 mg/mL) for precise CFU determination and determining the input ratio of Δ*lacZ* (white) to Δ*cvpA* (blue). 4 infant rabbits were inoculated and inoculated and monitored as described above, and colon samples collected. Tissue homogenate was plated on LB agar plates containing Gm and X-Gal (60 mg/mL) and CFU were counted the following day. A competitive index was calculated by dividing the burden of Δ*cvpA* divided by the Δ*lacZ* burden, adjusted for the input ratio.

### Δ*cvpA* complementation assays

*cvpA* was inserted into the expression vector pMMB207 [126] (ATCC #37809) downstream of the IPTG-inducible promoter using isothermal assembly. The resulting plasmid, pMMB207-*cvpA*, as well as an empty vector control, were transformed into Δ*cvpA*. To rescue the Δ*cvpA* DOC sensitivity phenotype, we used these strains in a bile salt sensitivity assay as described above. We found that expression from this plasmid was very leaky at basal (non-induced) conditions and could rescue the *cvpA* DOC sensitivity even without addition of IPTG.

### Computational analysis

To enable comprehensive functional/pathway analyses in EHEC we carried out BLAST-based comparisons between the old EHEC genome sequence and annotation system (NCBI Accession Numbers AE005174 and AF074613 [7]) and the new sequence and annotation system (NZ_CP008957.1 and NZ_CP008958.1 [8]) (S1 Table). This comparison links the new annotations (RS locus tags) to the original ‘Z numbers’ and their associated function and pathway annotation.

To make the correspondence table (S1 Table) between the old EHEC annotation system (Z Numbers) and the new system (RS Numbers), local nucleotide BLAST with output format 6 was used. First, a reference nucleotide database was generated from the newest EHEC sequence and annotation (NZ_CP008957.1 and NZ_CP008958.1). The EHEC genome sequence containing Z number annotations (AE005174 and AF074613) was used as the query. Of the 6032 EDL933 genes, 5508 have 85% or greater nucleotide identity to a Z Numbered locus. 4796 of these genes match only one Z Numbered locus and 712 genes match multiple Z Numbers. These cases are frequently due to repetitive genomic sequences (such as cryptic phage genes, transposons, or insertion elements) or situations cases where two loci have been merged into one locus. Due to gaps and ambiguity present in the original EDL933 sequence, we did not filter on alignment length in order to find the best matches. In cases where there was one matching locus, the alignment was always ≥90% of the length of the gene. For genes with multiple matches, the length of the alignment varied.

There are 524 genes with no corresponding Z Number, presumably loci that are newly recognized genes, and also 71 Z Numbers not assigned to an RS Number, which are loci now recognized as intergenic regions or not present in the final assembly.

To find the K-12 homolog for EHEC genes (S2 Table), local BLAST was also used. A reference nucleotide and amino acid database was generated from MG1655 K-12 (NC_000913.3), and the newest EHEC genome sequence was used as the query. For pseudogenes and genes coding for RNA, ≥90% nucleotide identity across ≥90% of the gene length was considered a homolog. For protein coding genes, ≥90% amino acid identity across ≥90% of the amino acid sequence was considered a homolog.

To find KEGG pathways and COG assignments for genes of interest, the Z correspondence table was used to look up the Z number of each gene. The Z number and corresponding functional information was searched on the EHEC KEGG database.

To determine if COGs were enriched in certain groups of genes (such as conditionally depleted genes), a COG enrichment index was calculated as in [56]. The COG Enrichment Index is the percentage of the genes of a certain category (essential genes or CD genes) assigned to a specific COG divided by the percentage of genes in that COG in the entire genome. A two-tailed Fisher’s exact test was used to determine if this ratio was independent of grouping. A Bonferroni correction was applied for multiple hypothesis testing. A p-value of <0.002 was considered to be significant.

Sequencing saturation of TIS libraries was determined by randomly sampling 100,000 reads from each library and identifying the number of unique mutants in that pool. Libraries are sequenced to saturation when no new mutants are identified as additional reads are added. 2–4 million reads are sufficient to capture the depth of libraries used here.

Several protein prediction programs (PSLPred, HHPred, Phobius, Phyre2) [127–129] were used to analyze the CvpA amino acid sequence. Protter [130] was used to compile information from several of these searches and generate a topological diagram. PRED-TAT [131] was used to search for tat-secretion signals in the list of CD genes.

## Supporting information

S1 Fig Sequencing saturation of TIS libraries. Reads were randomly sampled from each library and the percentage of TA sites disrupted in each randomly selected pool were plotted for the EDL933 library (A), MG1655 library (B), the inoculum library used to infect infant rabbits (C), and the libraries recovered from 7 rabbit colons (D-J).
(TIF)

S2 Fig Assessment of non-neutral EHEC EDL933 genes. (A) Non-neutral genes (defined by EL-ARTIST as either regional or underrepresented) by Clusters of Orthologous Groups (COG) classification. COG enrichment index (displayed as log_2_ enrichment) is calculated as defined in (51) as the percentage of the CD genes assigned to a specific COG divided by the percentage of genes in that COG in the entire genome. A two-tailed Fisher’s exact test with a Bonferroni correction was used to test the null hypothesis that enrichment is independent of TIS status. p-values considered to be significant if <0.002. Single asterisks (*) indicates p-value <0.002, double asterisks (**) indicate p-value <0.001, and triple asterisks (***) indicate p-value <0.0001. (B) GC content (%) of EDL933 genes classified as either neutral (blue) or non-neutral (regional + underrepresented; red) by TIS. Distributions are compared using a Mann-Whitney U non-parametric test; triple asterisks (***) indicate p-value of <0.0001. (C) GC content (%) of EDL933 genes classified as either having homologs in MG1655 (homolog) or lacking homologs (divergent). Distributions are compared using a Mann-Whitney U test; triple asterisks (***) indicate p-value of <0.0001. (D) TA insertions across *kdsC* in EDL933 (left) and MG1655 (right).
(TIF)

S3 Fig Con-ARTIST and CompTIS classification of genes important for colonization. (A-G) Distribution of percentage TA site disruption in libraries recovered from 7 rabbit colons. These distributions are overlaid with Con-ARTIST classification (queried, blue; CD (conditionally depleted), red) as described in Fig 2B. (H) Variance explained by each gene-level (gl) principal component for glPCA performed across the 7 rabbit screens. (I) Gene-level principal component 1 (glPC1) coefficients for each rabbit dataset. (J) Heatmap of the log_2_ fold change for each gene with a glPC1 score that falls within the bottom 10% of the distribution. Each column represents genes from a separate rabbit replicate. Genes are ordered by glPC1 score, lowest at the top of the heatmap and highest at the bottom.
(TIF)

S4 Fig In vitro growth and morphology of mutants. (A) 17 mutant strains plus the wild-type were grown in LB and turbidity measured by optical density. The average of three readings with the standard deviation is plotted. (B) Cell-shape defects of Δ*sufI*, Δ*envC*, Δ*tatABC*, and Δ*prc* mutants in high osmolality media. Morphology in LB (top) or LB supplemented with 0.3M NaCl (bottom) is shown.
(TIF)

S5 Fig Characterization of Δ*cvpA*. (A) Biofilm production in WT and Δ*cvpA* using crystal violet staining and absorption. Levels were normalized to a percent of the WT value; three samples were analyzed and the geometric means and geometric standard deviation are plotted. The differences between the two groups were not significant (n.s.) by Mann-Whitney U. (B) WT and Δ*cvpA* struck to single colonies on an agar plate made with YESCA media supplemented with Congo Red to detect curli fibers. (C) 1:1 competitive infection between Δ*cvpA* and Δ*lacZ*. (D) WT and Δ*cvpA* struck to single colonies on an agar plate containing minimal media with no exogenous purines. (E) Dilution series of *Vibrio cholerae* C6706 WT and *cvpA*::tn plated on LB and LB 1% deoxycholate (DOC). (F) Dilution series of *Vibrio parahaemolyticus* WT and Δ*cvpA* plated on LB and LB 1% deoxycholate (DOC).
(TIF)

S1 Table RS to Z annotation correspondance table. (XLSX)

S2 Table EL-ARTIST analysis of EHEC EDL933. (XLSX)

S3 Table KEGG pathways associated with EHEC non-neutral genes. (XLSX)

S4 Table EL-ARTIST analysis of *E*. *coli* K12 MG1655. (XLSX)

S5 Table Uniquely non-neutral genes in EHEC EDL933. (XLSX)

S6 Table Analysis of EHEC library passaged through seven rabbits using Con-ARTIST and CompTIS. (XLSX)

S7 Table KEGG pathways associated with EHEC genes required to colonize infant rabbits. (XLSX)

S8 Table Bacterial strains used in this study. (XLSX)

S9 Table Oligonucleotides and barcodes used in this study. (XLSX)
