# Supplementary figures and images for: Transposon-insertion sequencing screens unveil requirements for EHEC growth and intestinal colonization
Source: PLoS Pathog. 2019 Aug 12;15(8):e1007652. doi: 10.1371/journal.ppat.1007652 (PMC6705877; doi:10.1371/journal.ppat.1007652)

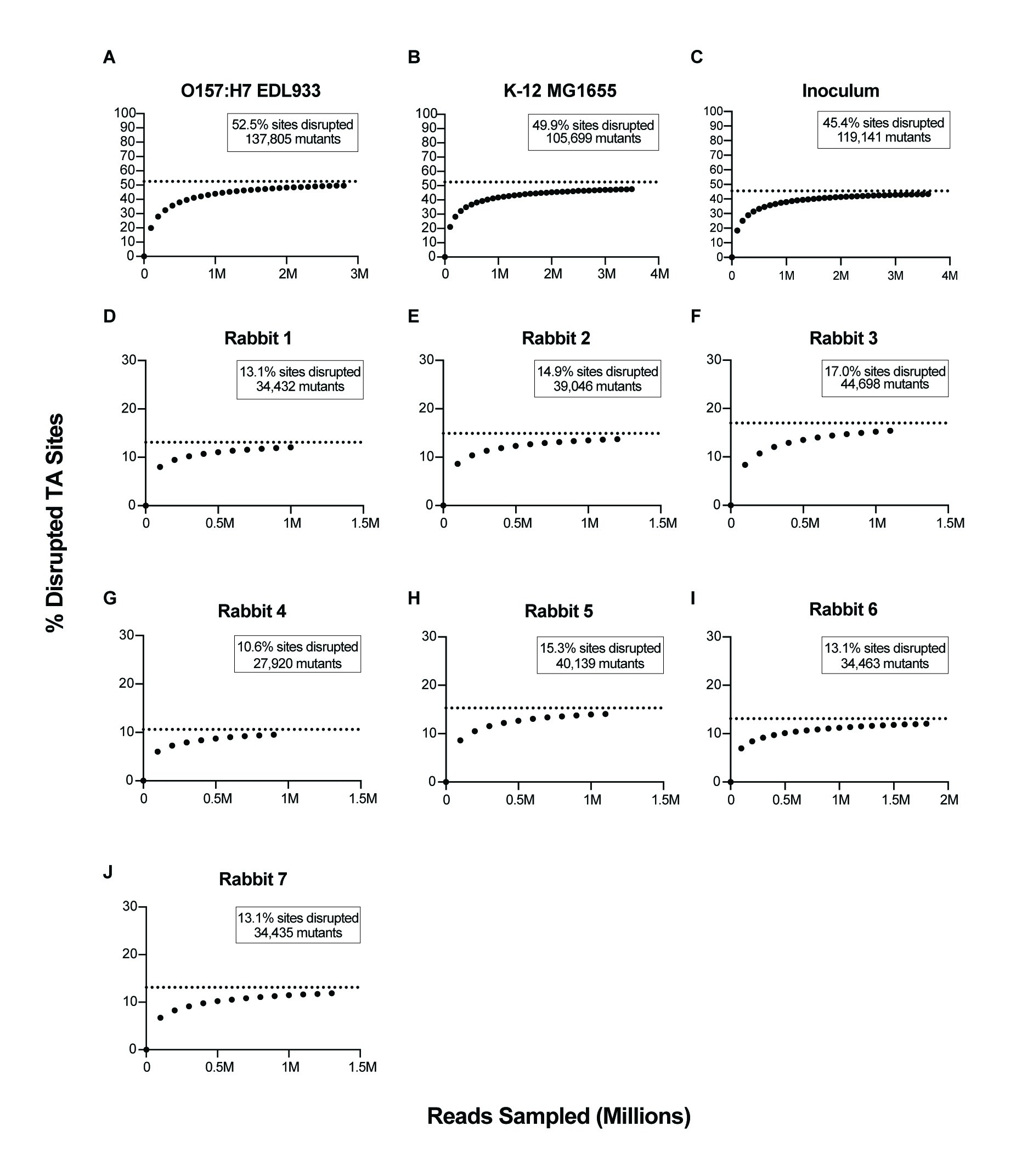

Supplement: S1 Fig — Reads were randomly sampled from each library and the percentage of TA sites disrupted in each randomly selected pool were plotted for the EDL933 library (A), MG1655 library (B), the inoculum library used to infect infant rabbits (C), and the libraries recovered from 7 rabbit colons (D-J). (TIF) [file ppat.1007652.s001.tif]

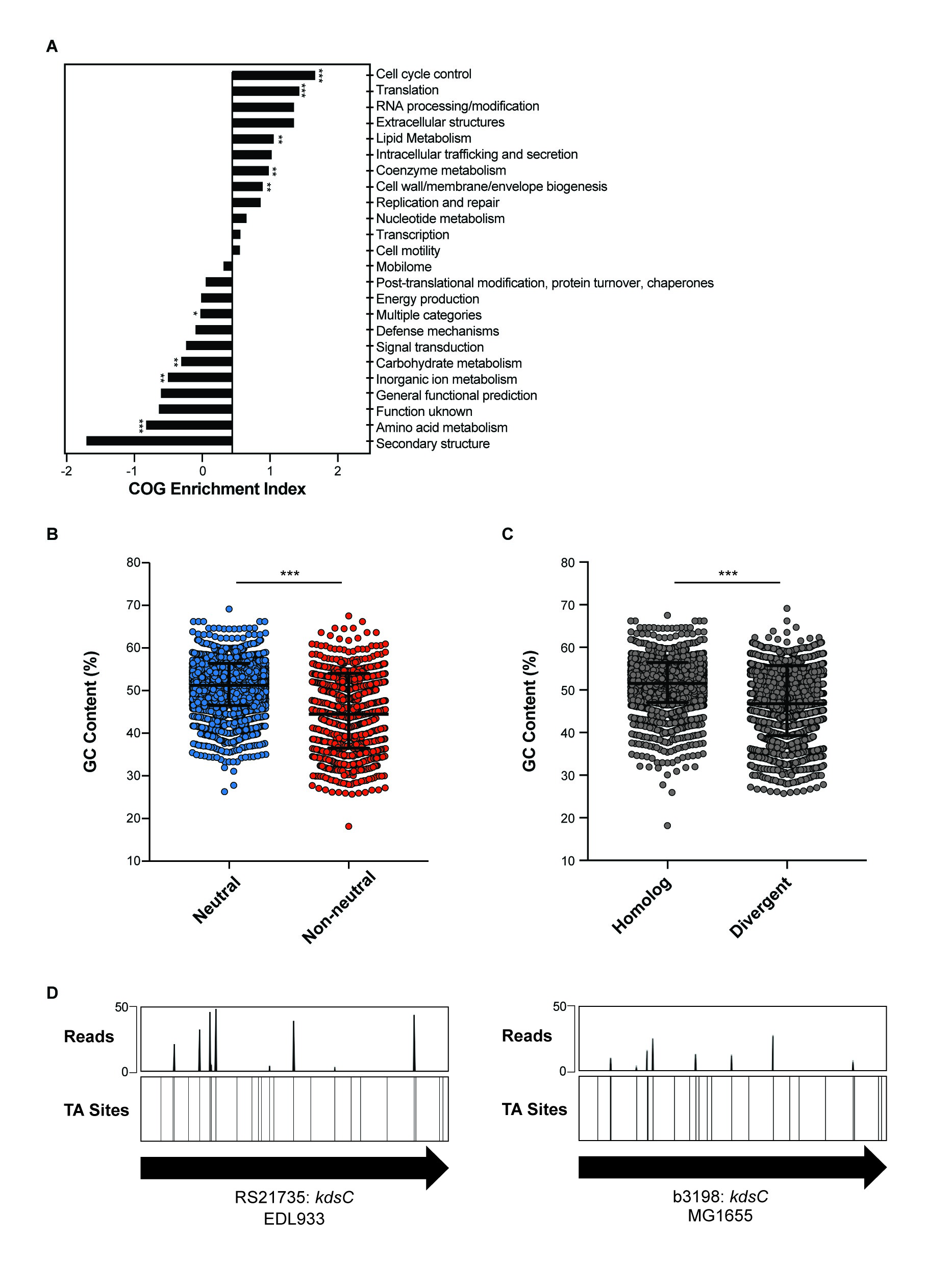

Supplement: S2 Fig — (A) Non-neutral genes (defined by EL-ARTIST as either regional or underrepresented) by Clusters of Orthologous Groups (COG) classification. COG enrichment index (displayed as log2 enrichment) is calculated as defined in (51) as the percentage of the CD genes assigned to a specific COG divided by the percentage of genes in that COG in the entire genome. A two-tailed Fisher’s exact test with a Bonferroni correction was used to test the null hypothesis that enrichment is independent of TIS status. p-values considered to be significant if <0.002. Single asterisks (*) indicates p-value <0.002, double asterisks (**) indicate p-value <0.001, and triple asterisks (***) indicate p-value <0.0001. (B) GC content (%) of EDL933 genes classified as either neutral (blue) or non-neutral (regional + underrepresented; red) by TIS. Distributions are compared using a Mann-Whitney U non-parametric test; triple asterisks (***) indicate p-value of <0.0001. (C) GC content (%) of EDL933 genes classified as either having homologs in MG1655 (homolog) or lacking homologs (divergent). Distributions are compared using a Mann-Whitney U test; triple asterisks (***) indicate p-value of <0.0001. (D) TA insertions across kdsC in EDL933 (left) and MG1655 (right). (TIF) [file ppat.1007652.s002.tif]

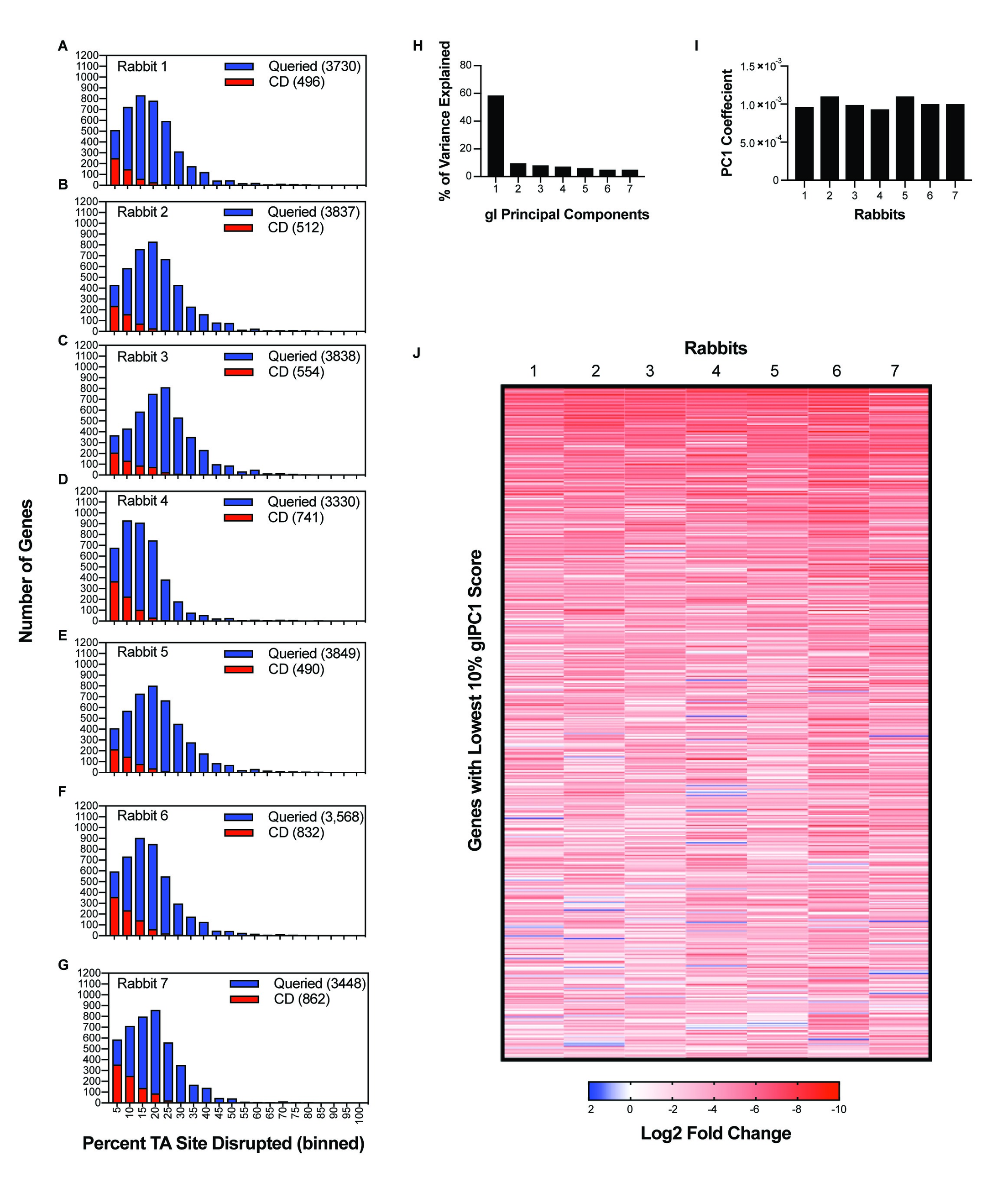

Supplement: S3 Fig — (A-G) Distribution of percentage TA site disruption in libraries recovered from 7 rabbit colons. These distributions are overlaid with Con-ARTIST classification (queried, blue; CD (conditionally depleted), red) as described in Fig 2B. (H) Variance explained by each gene-level (gl) principal component for glPCA performed across the 7 rabbit screens. (I) Gene-level principal component 1 (glPC1) coefficients for each rabbit dataset. (J) Heatmap of the log2 fold change for each gene with a glPC1 score that falls within the bottom 10% of the distribution. Each column represents genes from a separate rabbit replicate. Genes are ordered by glPC1 score, lowest at the top of the heatmap and highest at the bottom. (TIF) [file ppat.1007652.s003.tif]

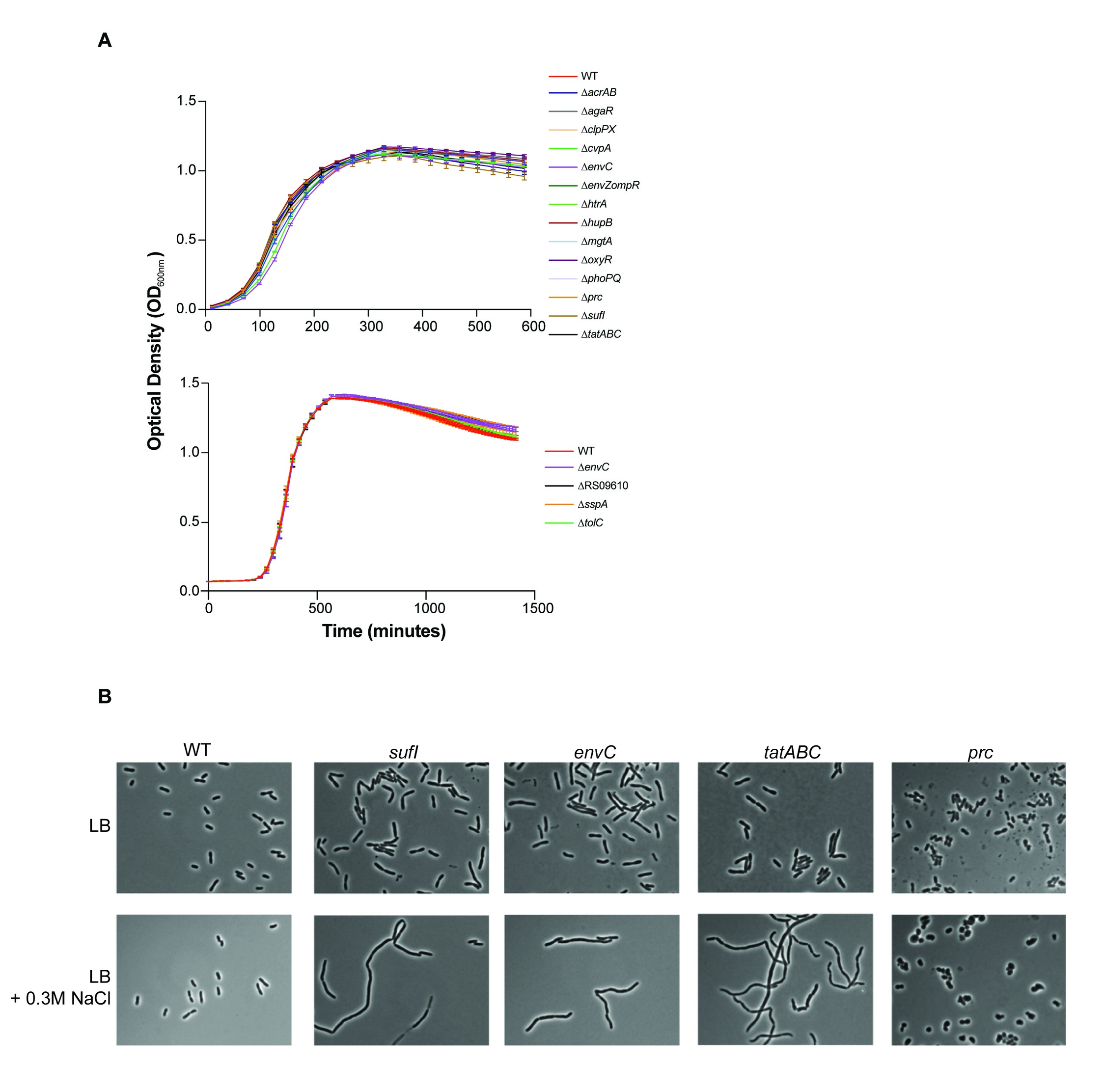

Supplement: S4 Fig — (A) 17 mutant strains plus the wild-type were grown in LB and turbidity measured by optical density. The average of three readings with the standard deviation is plotted. (B) Cell-shape defects of ΔsufI, ΔenvC, ΔtatABC, and Δprc mutants in high osmolality media. Morphology in LB (top) or LB supplemented with 0.3M NaCl (bottom) is shown. (TIF) [file ppat.1007652.s004.tif]

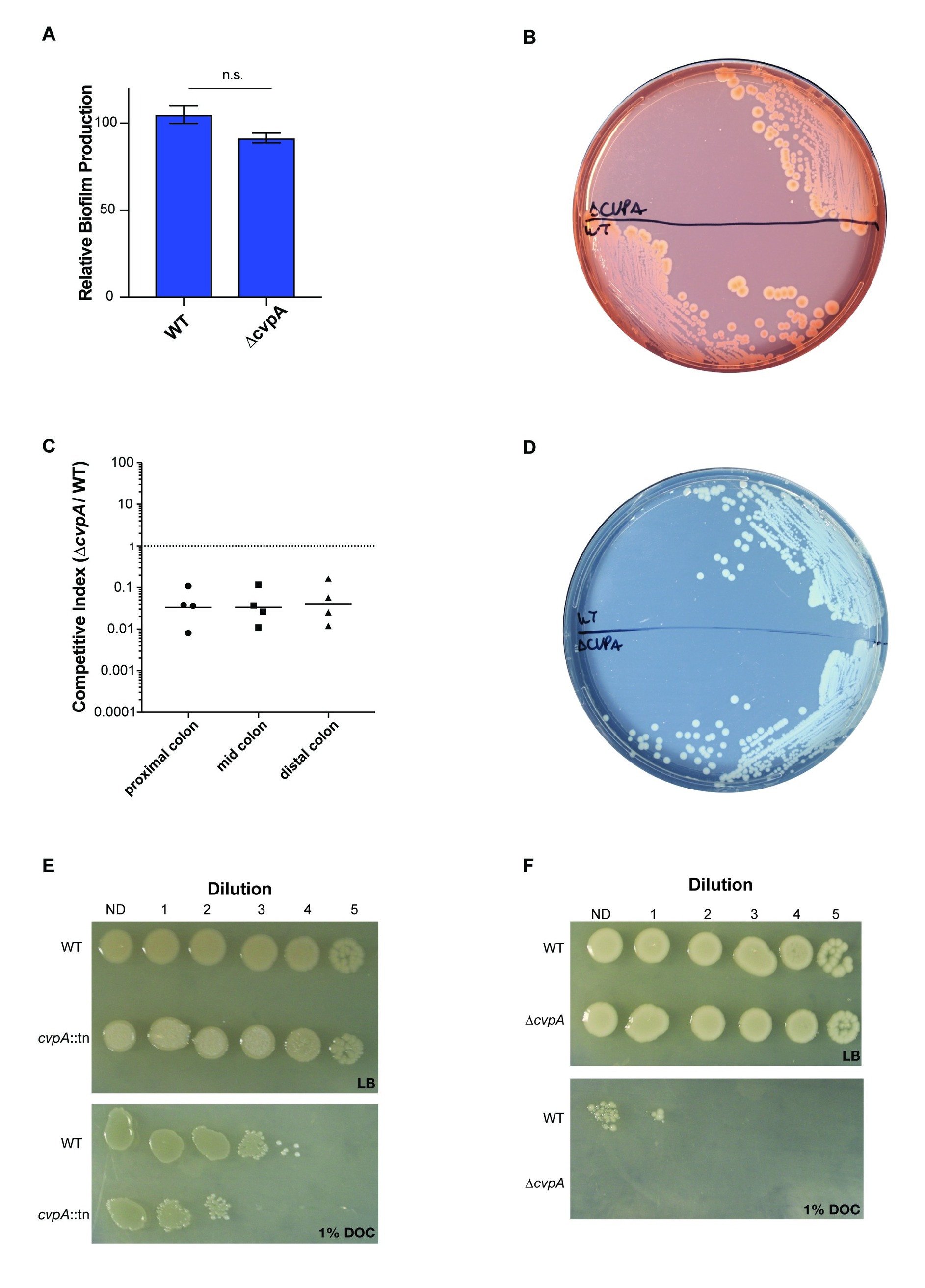

Supplement: S5 Fig — (A) Biofilm production in WT and ΔcvpA using crystal violet staining and absorption. Levels were normalized to a percent of the WT value; three samples were analyzed and the geometric means and geometric standard deviation are plotted. The differences between the two groups were not significant (n.s.) by Mann-Whitney U. (B) WT and ΔcvpA struck to single colonies on an agar plate made with YESCA media supplemented with Congo Red to detect curli fibers. (C) 1:1 competitive infection between ΔcvpA and ΔlacZ. (D) WT and ΔcvpA struck to single colonies on an agar plate containing minimal media with no exogenous purines. (E) Dilution series of Vibrio cholerae C6706 WT and cvpA::tn plated on LB and LB 1% deoxycholate (DOC). (F) Dilution series of Vibrio parahaemolyticus WT and ΔcvpA plated on LB and LB 1% deoxycholate (DOC). (TIF) [file ppat.1007652.s005.tif]
